# Photoisomerization of Azobenzene‐Extended Charybdotoxin for the Optical Control of K_v_1.2 Potassium Channel Activity

**DOI:** 10.1002/anie.202423278

**Published:** 2025-03-09

**Authors:** Yanis Achouba, Basile Peres, Steven Ascoët, Hervé Meudal, Cécile Caumes, Claude Zoukimian, Hugo Millet, Maureen Choteau‐Bodor, Cathy Carvalhosa, Mikael Croyal, Fella Bouchama, Heike Wulff, Stéphane Téletchéa, Rémy Béroud, Eléna Ishow, Céline Landon, Ahcène Boumendjel, Jérôme Montnach, Michel De Waard

**Affiliations:** ^1^ L'institut du thorax Nantes Université CNRS, INSERM Nantes F‐44000 France; ^2^ Département de Pharmacochimie Moléculaire Université Grenoble Alpes CNRS Grenoble F‐38000 France; ^3^ Center for Molecular Biophysics CNRS Orléans 45071 France; ^4^ Smartox Biotechnology Saint‐Egrève 38120 France; ^5^ SB‐Peptides Saint‐Egrève 38120 France; ^6^ Department of Pharmacology University of California Davis CA USA; ^7^ Nantes Université CNRS, U2SB, UMR 6286 Nantes F‐44000 France; ^8^ CEISAM Nantes Université CNRS Nantes 44322 France; ^9^ Université Grenoble Alpes La Tronche 38706 France; ^10^ Laboratory of Excellence «Ion Channels, Science and Therapeutics» Valbonne F‐06560 France

**Keywords:** Azobenzenes, Click chemistry, Ion channels, Photopharmacology, Photoswitches

## Abstract

Natural peptides from animal venoms effectively modulate ion channel activity. While photoswitches regulate small compound pharmacology, their application to natural peptides rich in disulfide bridges and active on ion channels is novel due to larger pharmacophores. A pilot study integrating azobenzene photoswitches into charybdotoxin (ChTx), known for blocking potassium channels is initiated. Two click‐chemistry‐compatible azobenzene are synthesized differing in length and amide orientation (Az_1_ & Az_2_). Az_1_ is grafted onto ChTx at various amino acid positions using L‐azidohomoalanine mutation. ChTx monomers outperformed dimers, particularly with azobenzene at position 14, by exhibiting optimal photoswitching activity. In the *cis* configuration, Az_1_ altered ChTx's pharmacophore, reducing potassium channel blockage, while conversely, Az_2_ increased ChTx potency. This study pioneers photoswitch application to complex peptides, leveraging structure‐activity relationships. Successful integration depends on precise azobenzene positioning and chemical grafting guided by SAR insights. This advancement underscores the adaptability of photoswitch technology to intricate peptide structures, offering new avenues for pharmacological modulation.

## Graphical abstract

Click‐chemistry compatible azobenzenes are synthesized and coupled on various positions at the surface of a disulfide‐bridged natural peptide. Monomers and dimers are produced and linked by azobenzene. The impact of azobenzene photoswitching on the ion channel blocking potency of the peptide is evaluated and revealed that proper positioning leads to successful photomodulation.

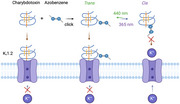

## Introduction

The primary objective of photopharmacology is to refine the specificity of drug action in vivo, limiting its impact on targeted tissues or cell types to mitigate unwanted side effects on non‐targeted organs.^[^ ^1^ ^]^ This spatial confinement of effects is complemented by temporal precision, as photopharmacology enables the regulation of drug activity according to defined timeframes. Achieving these advancements entails modifying drugs to be light‐sensitive, with chemical alterations tailored to specific photopharmacology technologies. Among these, the minimalist approach of “caging” compounds with photosensitive groups offers spatial and temporal control by releasing active drugs upon illumination. However, concerns about its irreversible nature have led to the development of several molecular photoswitches,^[^ ^2^, ^3^, ^4^, ^5^, ^6^, ^7^, ^8^, ^9^, ^10^, ^11^ ^]^ which dynamically toggle drug conformations between active and inactive states based on light wavelength.^[^ ^6^, ^12^, ^13^, ^14^ ^]^ While intellectually appealing, incorporating photoswitches or azologization modifications can alter a drug's potency for its target, posing challenges for practical application. Particularly with small compounds, photoswitching may significantly modify drug properties, complicating in vivo use and raising safety concerns for clinical applications.

To overcome some of these obstacles, researchers have explored hybrid approaches, combining photoswitching with genetic modifications of drug targets by tethering approaches.^[^ ^4^, ^15^, ^16^ ^]^ Another strategy consists of applying the photoswitching technology to larger compounds such as peptides with the hope that photoswitch incorporation has a minimal impact on activity and pharmacokinetics. Photoswitchable peptides have been developed on several occasions to control secondary structure formation,^[^ ^17^, ^18^, ^19^, ^20^, ^21^ ^]^ or to regulate biological functions^[^ ^22^ ^]^ such as antibiotic activity,^[^ ^23^ ^]^ enzymatic processes,^[^ ^23^, ^24^ ^]^ protein‐protein interactions,^[^ ^7^, ^25^ ^]^ integrins,^[^ ^26^ ^]^ and G protein‐coupled receptors.^[^ ^27^, ^28^ ^]^ Most of these examples involve small, simple peptides with few disulfide bridges. To our knowledge, none of the peptide‐based approaches used complex natural peptides aimed at modulating ion channels, which are fundamental to cellular excitability. Indeed, photoswitching larger biological compounds presents distinct challenges. Unlike small peptides, larger peptides with numerous disulfide bridges pose complexities in modifying them into photoswitchable ligands. Addressing these challenges involves integrating photoswitches without disrupting peptide structures and ensuring effective light modulation of larger pharmacophores.

Herein, as a case study, we focused on charybdotoxin (ChTx), a venom peptide known for its potent activity on K_v_ potassium channels, which folds via disulfide bridges.^[^ ^29^, ^30^ ^]^ Traditional coupling methods with maleimide‐containing azobenzenes (Az) were unsuitable, leading us to develop a first click‐chemistry compatible Az_1_ coupled to the peptide through strategic amino acid substitutions outside the pharmacophore. This Az‐scanning approach aimed to maintain peptide potency while preserving photoswitch impact on pharmacology. We also explored if ChTx dimers coupled with click chemistry‐compatible Az_1_ could enhance steric hindrance effects or not. Finally, we chemically synthesized and functionally validated, on the most relevant ChTx position, a new click‐compatible Az_2_ compound with better E‐to‐Z conversion. Our findings demonstrate successful post‐folding coupling of the two Az to ChTx, largely preserving peptide pharmacology. Photoswitching modulated peptide potency for K_v_1.2 channels, with outcomes influenced by coupling positions, dimer formation, and the chemical nature of the Az grafted. This pilot study demonstrates that the photoswitching technology can be successfully adapted to peptides, folded with multiple disulfide bridges, and active on ion channels while preserving their exceptionally high potency.

## Results and Discussion

### Photochemical Properties of Az_1_

**Figure 1:**
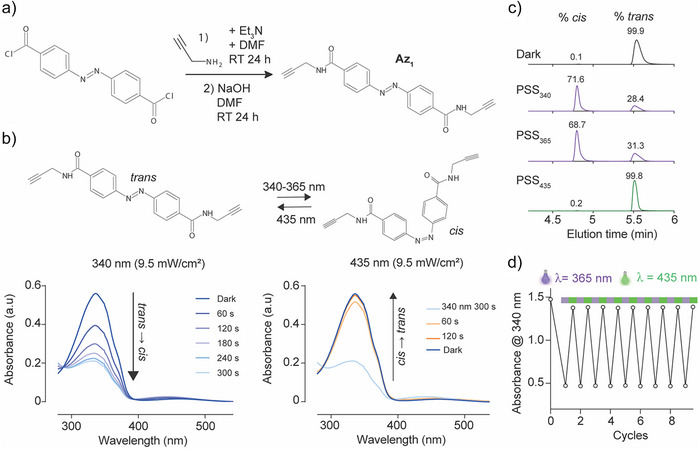
Chemical properties of a click chemistry‐compatible azobenzene (Az_1_). a) Synthesis pathway of Az_1_. b) Isomerization reaction of Az_1_ promoted by UV light (*trans* to *cis*) and visible light (*cis* to *trans*) irradiation at a 9.5 mW cm^−2^ light intensity in 100% DMSO. Amide bonds are all in the *trans* configuration. c) Photostationary states of Az_1_ in the dark, after illumination at 365 or 340 nm and upon back‐switching by illumination at 435 nm as assessed by HPLC. d) Cycles of illuminations to probe the Az_1_ fatigue.

To produce a click‐compatible azobenzene product, (E)‐4,4′‐(diazene‐1,2‐diyl)bis(N‐(prop‐2‐yn‐1‐yl)benzamide) (Az_1_) was synthesized as described in the Experimental Section (Figure 1a) with a synthesis yield of 25%. ^1^H NMR, ^13^C NMR and HRMS analyses of Az_1_ are provided before and after photoswitching (Figure S1). Before irradiation, Az_1_ was thermally adapted and contained almost pure *trans*‐isomer with maximum absorption at λ_max_ = 340 nm. Irradiation at λ = 340 nm (9.5 mW cm^−2^) results in the formation of the *cis* isomer that saturates at 300 s (Figure 1b). A similar finding was obtained if irradiation was performed at λ = 365 nm (Figure S2), allowing us to use this wavelength which was suited to our functional studies. Irradiation at 435 nm (9.5 mW cm^−2^) for reverse‐switching allows for the fast recovery of a *trans*‐enriched compound (within ≈60 s). Photostationnary states (PSS) of both *cis*‐enriched and *trans*‐enriched solutions of Az_1_ illustrate that a maximum of 0.2% *cis* isomer is present for initial solutions stored in the dark and in the PPS_435_, while a maximum of 68.7% *cis* isomer can be reached after 365 nm illumination, only marginally increased to 71.6% if 340 nm is used instead of 365 nm (Figure 1c). The high PPS_435_ value of 99.8% was not due to thermal relaxation since the *cis* isomer was remarkably stable (Figure S3). No significant fatigue upon 10 cycles of illumination was observed (Figure 1d). Despite its incomplete transitions, Az_1_ can be used as a first scanning compound to investigate which position onto ChTx provides the best photoswitching results on ChTx potency and whether monomers are better than dimers, or vice versa. It also represents an opportunity to evaluate the impact of Huisgen cycloaddition on photoswitch efficiency.

### Strategy for Production of Optimal Az_1_‐coupled ChTx

**Figure 2:**
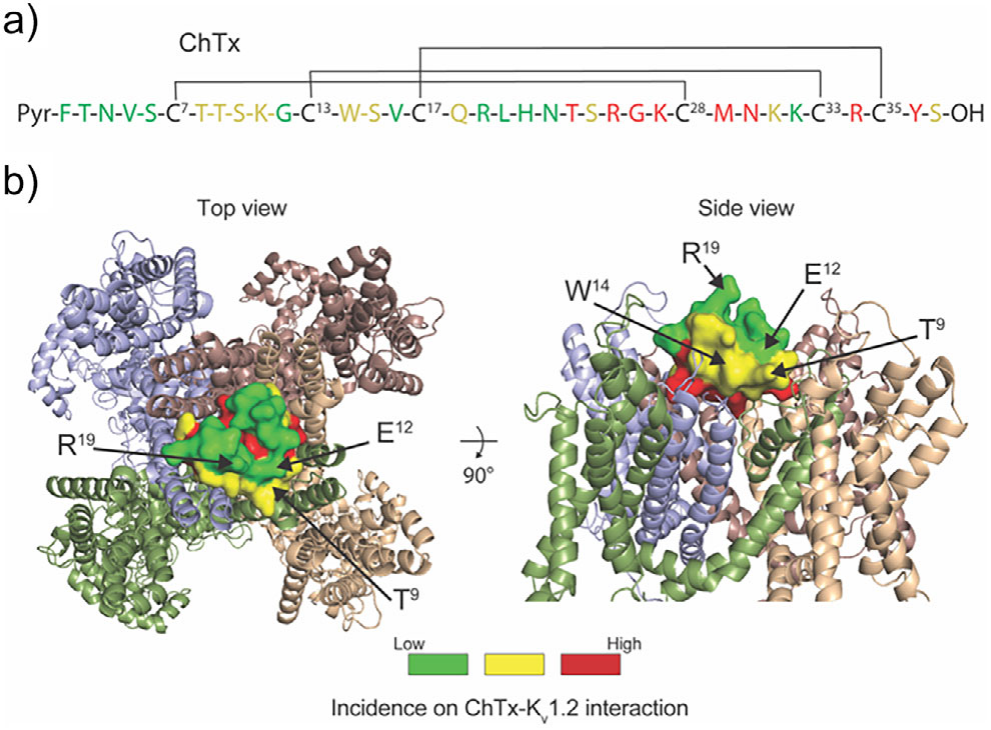
Strategy for the positioning of Ah substitution on ChTx. a) Primary structure of ChTx illustrating the disulfide bridging pattern and the critical residues of the pharmacophore (in red). Residues in yellow and green are defined as in (b) depending on location compared to the pharmacophore. b) 3D‐structure of K_v_1.2 channel with docked ChTx on the ionic pore in top and side views. The pharmacophore of ChTx is highlighted in red. Yellow residues of ChTx are in close proximity to K_v_1.2 structures, while green residues are those that are the most distant from the channel amino acid residues. The position of the residues to be substituted individually by Ah are illustrated (T^9^ and W^14^ from the yellow category, closest to the channel structure, and E^12^ and R^19^ from the green category).

ChTx, like most natural peptides originating from animal venoms, is a disulfide‐rich peptide. With a length of 37 amino acids, it contains three disulfide bridges that fold according to the most thermodynamically favorable pattern C^7^‐C^28^, C^13^‐C^33,^ and C^17^‐C^35^ (Figure 2a). This is the sole disulfide bridge pattern that produces a pharmacologically active peptide conformation. The addition of sterically bulky chemical moiety anywhere on the sequence (like Az_1_), greatly enhances the risk of misfolding (acquisition of an inappropriate disulfide bridge pattern and/or loss of secondary structures). Knowing that 15 theoretical disulfide bridge patterns are possible upon folding, this risk has to be minimized by i) first, synthesizing click chemistry‐compatible ChTx mutated analogs that fold properly, ii) second, ensuring that these analogs remain pharmacologically active, and iii) third, grafting the Az_1_ compound by click chemistry onto those folded/active ChTx analogs. This post‐folding strategy of Az_1_ extension has thus been chosen over the pre‐folding one in which Az_1_ is grafted onto the linear peptide because it is inherently safer and should produce acceptable yields. It was pioneered by us on a various number of complex natural peptides.^[^ ^31^, ^32^, ^33^, ^34^ ^]^ Thus, first, we aimed at establishing the optimal position where to graft Az_1_ onto ChTx–in an Az_1_ scanning approach logic – without excessively interfering with peptide potency for its target. By using the 3D structure of the complex between ChTx and the K_v_1.2 potassium channel^[^ ^30^ ^]^ (PDB: 4JTA) and previous extensive structure‐function relationship study that defines the pharmacophore of ChTx,^[^ ^29^ ^]^ we defined three regions of the ChTx structure according to the risk of perturbing the interaction upon residue mutation and Az_1_ grafting (Figure 2b). The region in red is the pharmacophore of ChTx and is formed by residues in direct interaction with K_v_1.2. Mutation of the pharmacophore and addition of Az_1_ in this region would dramatically affect the potency of the peptide, regardless of Az_1_ isomeric conformation, and was therefore avoided. The region in green is opposite to the pharmacophore and mutation of this region is the least likely to affect ChTx potency for K_v_1.2. Therefore, grafting of Az_1_ in this region should preserve good binding interaction with K_v_1.2 but may limit the functional impact of Az_1_ photoswitching. The region in yellow is conceptually and theoretically the most interesting one for mutation and Az_1_ grafting. Coupling of Az_1_ in this region limits the risk of peptide potency alteration by itself but increases the probability that Az_1_ photoswitching perturbs ChTx pore‐blocking efficacy.

To keep in line with these considerations, we, therefore, decided to produce four analogs of ChTx, two mutated in the green domain (E^12^ and R^19^) and two others in the yellow domain (T^9^ and W^14^) (Figure 2b). As for amino acid substitutions, we chose L‐azidohomoalanine (Ah) to replace the native residues since it possesses a short lateral chain on which click chemistry can be performed with Az_1_.

### Chemical Syntheses and Functional Evaluation of ChTx‐Ah Analogues on K_v_1.2‐Expressing Cells

Fmoc‐based solid phase peptide chemistry was used to synthesize each of the click chemistry‐compatible ChTx‐Ah analogs. Folding of the crude synthetic peptides was performed in 0.1 m Tris buffer at pH 8.0 in the presence of the redox couple GSH/GSSG. After folding/oxidation, the peptides were purified to homogeneity, and their proper masses were verified by LC‐ESI QTOF (Figure S4). No folding issues were observed which is coherent with minor sequence modifications of ChTx when it comes to mutagenesis. The potency of the four new ChTx‐Ah analogs to inhibit K^+^ current from L929 cells stably overexpressing mouse K_v_1.2 channels was assessed by a high‐throughput automated patch‐clamp system (Figure S5). The data indicate a 4‐fold blocking potency gain for ChTx‐Ah^12^ (IC_50_ = 1.2 nm instead of 5 nm), preservation of potency for ChTx‐Ah^9^ (IC_50_ = 6.3 nm), or a mild twofold reduction of potency for ChTx‐Ah^19^ and ChTx‐Ah^14^ (IC_50_ = 11.5 and 9.8 nm). These results confirm that the Ah‐substitutions spared the major pharmacophore residues required for the high potency of ChTx.

### Chemical Syntheses of ChTx‐Ah‐Az_1_ Monomers and ChTx‐Ah‐Az_1_‐Ah‐ChTx Dimers

**Figure 3:**
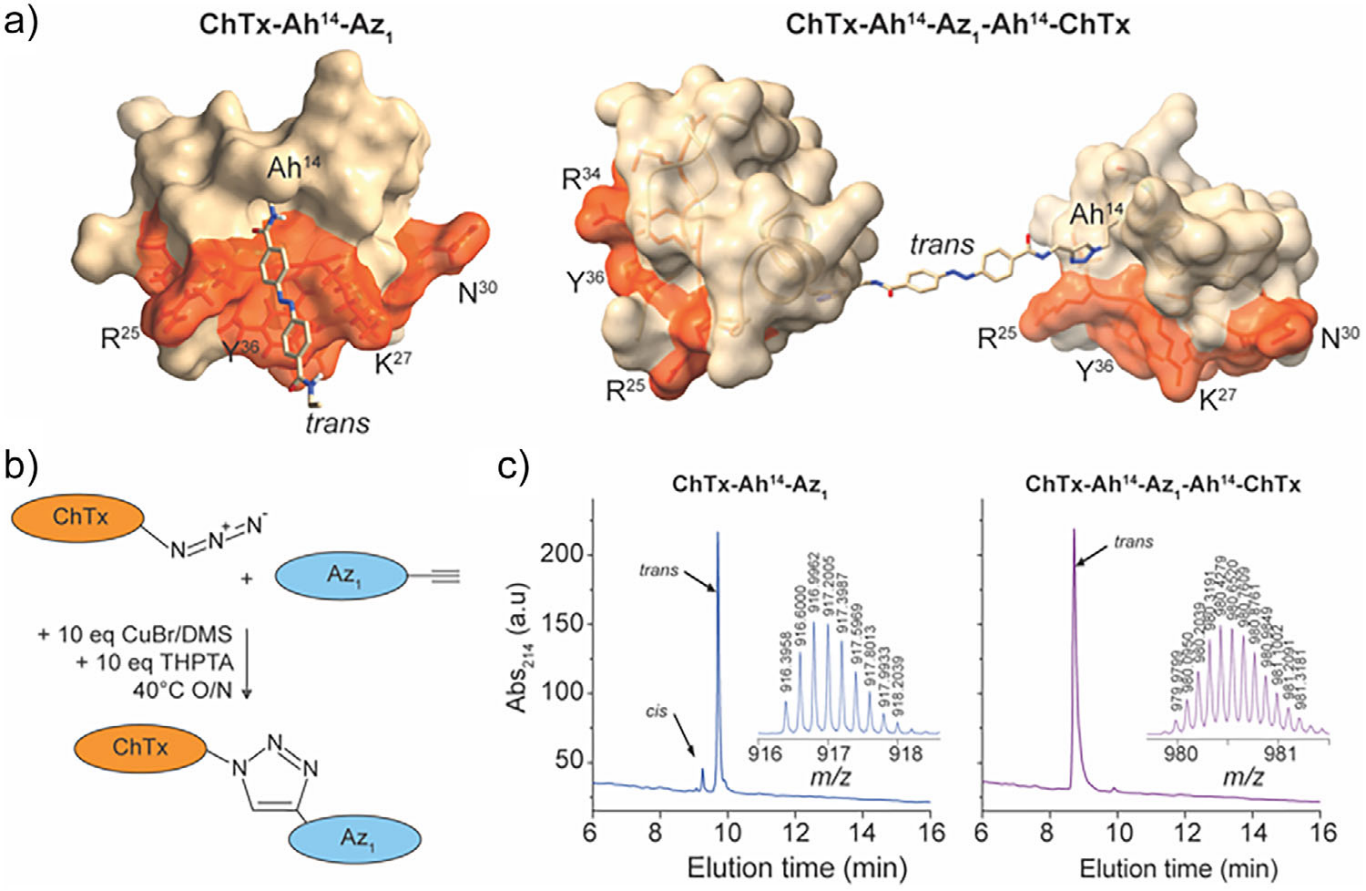
Production and purification of the ChTx‐Ah^14^‐Az_1_ monomer and the ChTx‐Ah^14^‐Az_1_‐Ah^14^‐ChTx dimer. a) Schematic 3D representation of the monomer and the dimer. The pharmacophore is given in red with K^27^ being the residue that plunges into the K_v_1.2 channel pore. The pharmacophore comprises T^23^, R^25^, G^26^, M^28^, N^29^, R^34^ and Y^36^ besides K^27^. The size and length of the Az_1_ linker in the *trans* configuration are proportionate. b) Click chemistry conjugation between ChTx‐Ah and Az_1_. c) Left panel: elution profile and [M + 5H]^5+^ MS (inset) of ChTx‐Ah^14^‐Az_1_ monomer after click chemistry conjugation in the 0.33 Az_1_/1 ChTx‐Ah^14^ stochiometric ratio. A small fraction of the *cis* isomer is also visible. Right panel: elution profile and [M + 9H]^9+^ MS of ChTx‐Ah^14^‐Az_1_‐Ah^14^‐ChTx dimer after click chemistry conjugation in the 3 Az_1_/1 ChTx‐Ah^14^ stochiometric ratio. The *cis* isomer is not detectable possibly because less stable in the case of the dimer.

Click chemistry conjugation of Az_1_ to Ah‐mutated ChTx was performed using two different molar ratios in order to favor the formation of either ChTx‐Ah‐Az_1_ monomers (3 ChTx‐Ah:1 Az_1_ ratio) or ChTx‐Ah‐Az_1_‐Ah‐ChTx dimers (1:3 ratio). Peptide concentrations used were 15 mm in a buffer that was compatible with both Ah‐mutated ChTx analogs and Az_1_. All compounds were purified by RP‐HPLC. For illustration, the position of Az_1_ in its thermally stable *trans* configuration coming out of ChTx at position 14, after clicking Az_1_ onto ChTx‐Ah^14^, is shown, for both monomer and dimer (Figure 3a). The click chemistry linking Az_1_ to ChTx‐Ah^14^ for both the monomer and the dimer is shown schematically (Figure 3b). Importantly, ChTx‐Ah^14^‐Az_1_ was stable for at least 6 h upon incubation with 10 mm GSH, indicating that such an azo‐extended peptide is compatible with in vivo experiments (Figure S6). In dark conditions, ChTx‐Ah^14^‐Az_1_ is thermally adapted and is composed of 95% *trans* isomer and 5% *cis* isomer. In contrast, the *cis*‐isomer of ChTx‐Ah^14^‐Az_1_‐Ah^14^‐ChTx dimer is not detectable showing that close to 100% *trans* isomers are present in dark storage conditions (Figure 3c). Regarding the other monomers, a similar trend of the presence of *cis‐*isomers was observed: 5% for ChTx‐Ah^9^‐Az_1_, 8% for ChTx‐Ah^12^‐Az_1_, and 9% for ChTx‐Ah^19^‐Az_1_ (Figure S7). Conversely, the *cis* isomer was hardly detected for one dimer (ChTx‐Ah^19^‐Az_1_‐Ah^19^‐ChTx), while it remained low (<6%) for ChTx‐Ah^12^‐Az_1_‐Ah^12^‐ChTx and ChTx‐Ah^9^‐Az_1_‐Ah^9^‐ChTx (Figure S7).

### Photo‐Isomerization Properties of the Monomers and Dimers

**Figure 4:**
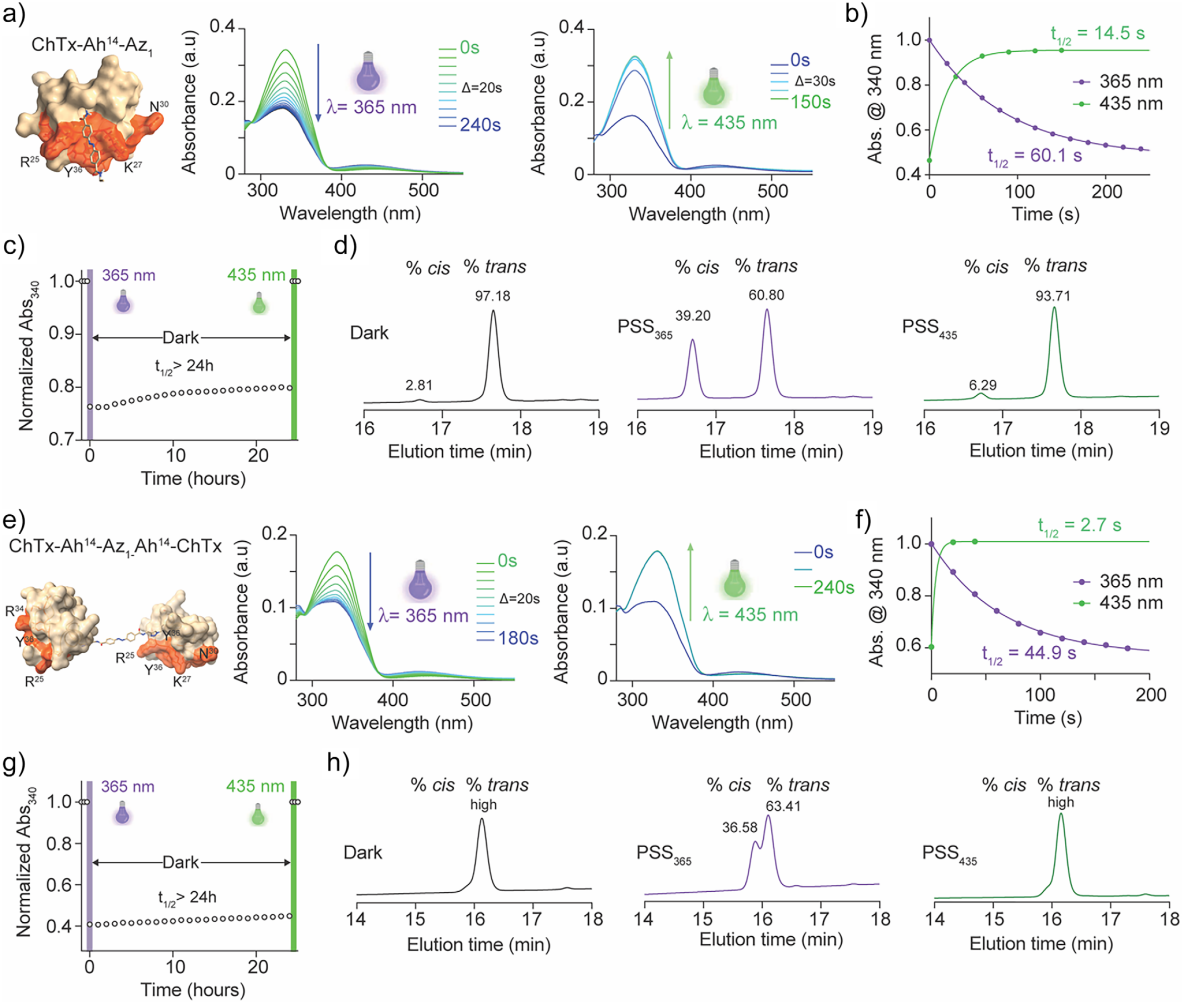
Isomerization properties of the ChTx‐Ah^14^‐Az_1_ monomer and ChTx‐Ah^14^‐Az_1_‐Ah^14^‐ChTx dimer. a) the kinetics of isomerization of the ChTx‐Ah^14^‐Az_1_ monomer, schematized on the left panel. Purple color represents *cis* isomer, while green represents *trans* isomer. b) Kinetics of the isomerization. Half‐isomerization times (t_1/2_) were extracted. c) Stability of the *cis* conformation of the ChTx‐Ah^14^‐Az_1_ monomer over a period of 24 h. d) Photostationary states of ChTx‐Ah^14^‐Az_1_ in the dark, after illumination at 365 nm and upon back‐switching by illumination at 435 nm, as assessed by HPLC. The y‐axis is the absorbance at 214 nm in arbitrary units. e–h) as for a–d) but for the ChTx‐Ah^14^‐Az_1_‐Ah^14^‐ChTx dimer. The absorbance for the dimer in (e) is about half the absorbance value observed in (a) for the monomer because a similar concentration of peptide was used in both conditions which implies half a concentration of Az_1_ in the dimer. In (h) the exact percentage of *cis* in dark and PSS_435_ conditions cannot be determined because of the proximity of the elution peaks. All peptide/Az_1_ conjugates are dissolved in water.

Next, we evaluated for all monomers and dimers the *trans*‐*cis* isomerization reactions under various wavelengths of illumination. Illuminating the ChTx‐Ah^14^‐Az_1_ monomer at λ = 365 nm at 9.5 mW cm^−2^, to favor the *cis* configuration, yielded a significant time‐dependent change in the absorbance properties of the peptide, characterized by a decrease of the absorbance band maximum centered at 340 nm (Figure 4a).

Conversely, subsequent illumination of the solution at 435 nm at the same intensity of 9.5 mW cm^−2^ resulted in the reverse switching to the *trans* configuration (Figure 4a). Reaction half‐life values at t_1/2_ were found to be 60.1 and 14.5 s for the *trans* to *cis* and the *cis* to *trans* isomerization, respectively (Figure 4b). Therefore, back‐switching to *trans* was 4.1‐fold faster than the *trans*‐to‐*cis* transformation. As Az_1_ was found stable in the *cis* configuration (Figure S3), we also measured the thermal stability of the Az_1_‐extended peptides. As shown, once the *cis* configuration has been induced, very limited back switching was observed in the dark for a period exceeding 24 h (Figure 4c). Illumination of the solution at 435 nm back‐switched ChTx‐Ah^14^‐Az_1_ to the *trans* configuration. Compositions of the solutions in their photostationary states were assessed by HPLC at 214 nm to get an idea of the percentage of conversions (Figure 4d). At this wavelength, the absorption stems from the peptide and is not affected by isomerization (Figure S8). In dark storage conditions, ≈2.8% *cis* was present, whereas illumination at λ = 340 nm at 9.5 mW cm^−2^ elevated this value to 39.2%. Reverse light‐switching restored a lower level of *cis* isomer down to 6.3%. The percentage of *cis* configuration reached with the ChTx‐Ah^14^‐Az_1_ monomer was not much different for the three other monomers with a value of 43.1% for ChTx‐Ah^12^‐Az_1_, being the best monomer (Figure S9). These observations indicate that the efficiency in producing the *cis* isomer is peptide‐position independent in the monomers. Also, the isomerization efficiency, being lower in the monomer compared to Az_1_ alone (68.7% *cis*, Figure 1), indicates that the azide‐alkyne cycloaddition may have altered the Az_1_ isomerization properties.

We next investigated the isomerization properties of the ChTx‐Ah^14^‐Az_1_‐Ah^14^‐ChTx dimer by following the kinetics of *trans* to *cis* (λ = 340 nm at 9.5 mW cm^−2^) and *cis* to *trans* (λ = 435 nm at 9.5 mW cm^−2^) isomerization (Figure 4e). Globally, saturation of the photochemical transformation occurred faster than with the ChTx‐Ah^14^‐Az_1_ monomer. Half‐lives of t_1/2_ = 44.9 s (*trans* to *cis* favored transition) and t_1/2_ = 2.7 s (*cis* to *trans*) were calculated for each of these isomerization reactions (Figure 4f). Here, the difference in the *trans* to *cis* and *cis* to *trans* kinetics was larger than that for the monomer with a 16.6‐fold faster reverse‐switching to the *trans* configuration (compared to 4.1‐fold for the monomer). While establishing the *cis* configuration was only 1.34‐fold faster for the dimer compared to the monomer, the reverse‐switching to *trans* was 5.37‐fold faster, indicating that back‐switching mainly accounts for the difference between the monomer and dimer. One interpretation would be that enhanced steric hindrance produced by two bulky peptides coming into close contact in the *cis* configuration accounts for a faster reverse‐switching of the dimer. It would however require a formal demonstration. Also, stress effects can be evoked since the two ends of Az_1_ are linked to a ChTx moiety. Despite this observation, we nevertheless noticed that the *cis* configuration of the dimer, like its monomer counterpart, remained remarkably stable in the dark for periods exceeding 24 h (Figure 4g). Again, photostationary states were measured by HPLC at 214 nm (Figure 4h). Because of the very close elution profiles of the *cis* and *trans* isomers, estimates of low percentages of *cis* isomers were too imprecise. However, after favoring the *cis* isomer, a total of 36.6% could be quantified. The isomerization properties of the three other dimers were also investigated (Figure S10). The *trans*‐to‐*cis* isomerization for the three other dimers was slightly faster than for ChTx‐Ah^14^‐Az_1_‐Ah^14^‐ChTx, the fastest being the ChTx‐Ah^19^‐Az_1_‐Ah^19^‐ChTx with t_1/2_ = 31.3 s. Also, the fastest *cis*‐to‐*trans* isomerization was carried by the ChTx‐Ah^9^‐Az_1_‐Ah^9^‐ChTx dimer (t_1/2_ = 2.7 s). However, photostationary state measurements indicate a remarkably stable value for the *cis* isomer in all dimers (close to 36%).

Several general conclusions can be drawn. First, *trans*‐to‐*cis* photoisomerization always took more time than *cis*‐to‐*trans* photoisomerization, regardless of whether we were dealing with a monomer or a dimer (Table 1). This difference may be due to dissimilarities in quantum yields in favor of a more efficient Z‐to‐E conversion or because of differential overlaps of the E/Z‐isomer absorption bands. Second, the position of Az_1_ onto ChTx had little influence on the kinetics of the *trans* to *cis* transformation regardless of whether we are dealing with a monomer or a dimer. The fastest *trans* to *cis* isomerization was for the ChTx‐Ah^19^‐Az_1_‐Ah^19^‐ChTx dimer with t_1/2_ = 31.3 s (Figure S10j), while the slowest was for the ChTx‐Ah^9^‐Az_1_‐Ah^9^‐ChTx dimer with t_1/2_ = 62.1 s (Figure S10b). The greatest kinetic difference between the monomers and the dimers was for the W^14^ mutation (1.34‐fold) and the lowest for the T^9^ mutation (1.01‐fold). Third, the greatest variations in kinetics were observed for the dimers. The fastest *cis*‐to‐*trans* reaction was found for the ChTx‐Ah^19^‐Az_1_ monomer with t_1/2_ = 2.1 s, whereas the slowest one was for the ChTx‐Ah^9^‐Az_1_ monomer with t_1/2_ = 14.9 s. Finally, it is worth noting that the acceleration of *cis* to *trans* transformation observed with the ChTx‐Ah^14^‐Az_1_‐Ah^14^‐ChTx dimer compared to the monomer is no longer true for the three other positions. These results seem to indicate that Az_1_ grafting at position 14 of ChTx provides interesting differences in isomerization behavior.

**Table 1:** Half isomerization times of Az_1_‐coupled ChTx monomers and dimers.

	t_1/2_ *trans → cis* [95% CI]	t_1/2_ *cis → trans* [95% CI]	Ratio
ChTx‐Ah^9^‐Az_1_	61.2 s [59.3–63.1]	14.9 s [13.9–15.9]	4.1
ChTx‐Ah^12^‐Az_1_	39.4 s [28.7–58.4]	11.4 s [7.8–15.3]	3.5
ChTx‐Ah^14^‐Az_1_	60.1 s [58.4–61.8]	14.5 s [13.9–14.9]	4.1
ChTx‐Ah^19^‐Az_1_	32.1 s [30.5–33.8]	2.1 s	15.3
ChTx‐Ah^9^‐Az_1_‐Ah^9^‐ChTx	62.1 s [57.9–66.7]	12.3 s [12.1–12.4]	5.0
ChTx‐Ah^12^‐Az_1_‐Ah^12^‐ChTx	34.8 s [30.8–39.8]	14.9 s [7.4–33.8]	2.3
ChTx‐Ah^14^‐Az_1_‐Ah^14^‐ChTx	44.9 s [42.1–48.2]	2.7 s	16.6
ChTx‐Ah^19^‐Az_1_‐Ah^19^‐ChTx	31.3 s [29.9–32.7]	7.2 s [5.1–9.3]	4.3

### Structural Consequences of Az_1_ Isomerization on ChTx

**Figure 5:**
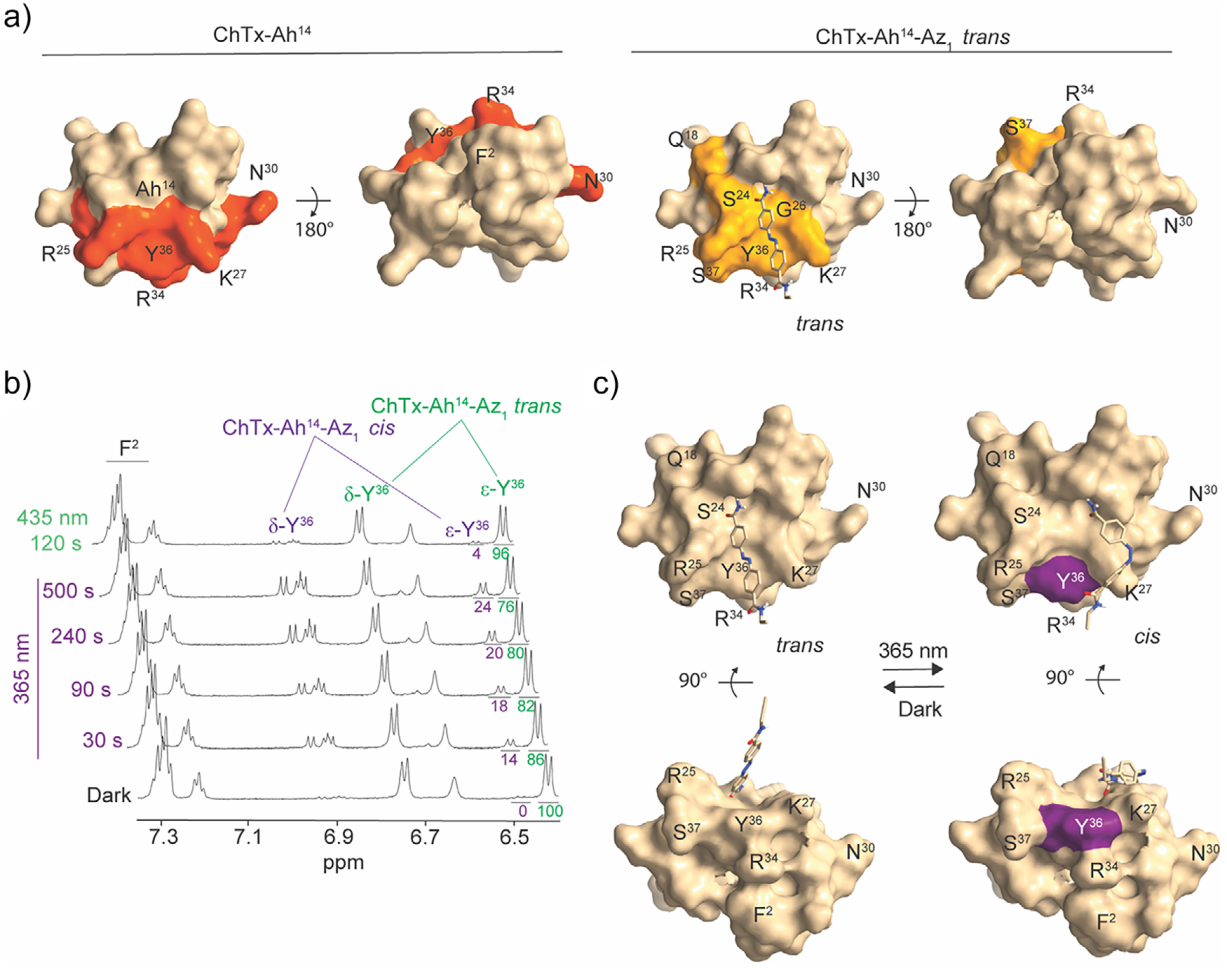
Structural consequences of Az_1_ cycloaddition onto ChTx‐Ah^14^ and *trans* → *cis* isomerization. a) Comparison of ChTx‐Ah^14^ and ChTx‐Ah^14^‐Az_1_ 3D structures. Variations in lateral chain positions were observed for Q^18^, S^24^, R^25^, G^26^, K^27^, Y^36^ and S^37^ upon Az_1_ attachment (in yellow, right panel). The position of the pharmacophore is shown for comparison on ChTx‐Ah^14^ alone (red color, left panel). b) 1D ^1^H‐NMR spectra illustrating the time‐dependent evolution of d‐Y^36^ and the e‐Y^36^ signals upon illumination at 365 nm of ChTx‐Ah^14^‐Az_1_, a signature of the *cis* conformation, and upon illumination at 435 nm or upon back thermal relaxation over 24 h in the dark. The incomplete back thermal relaxation is coherent with the data in Figure 4c. c) Peptide models illustrating the Az_1_ reorganization at the surface of ChTx‐Ah^14^‐Az_1_ upon *trans* to *cis* switching. In the *cis* configuration of Az_1_, the free end orients itself toward Y^36^ (purple color in *cis*). F^2^ position is not affected by the isomerization. Drawn by ChimeraX.

According to our observations, the *trans*‐to‐*cis* isomerization of Az_1_ coupled to ChTx peptide is measurable. We took advantage of having a significant level of isomerization to examine the consequences of i) Az_1_ coupling and ii) isomerization onto the ChTx structure (Figure 5). Earlier observations had shown that the covalent attachment of an orthogonal *o*‐nitroveratryloxycarbonyl (Nvoc) protecting group on the lateral chain of a Lys residue of a peptide, that is also stringently structured by a pattern of disulfide bridges, will bring in displacements in the exact position of the lateral chains of neighboring residues.^[^ ^35^, ^36^ ^]^ Something similar was thus expected upon the cycloaddition of Az_1_ to ChTx‐Ah^14^. Using 1D ^1^H NMR spectroscopy, Hα protons of *trans* ChTx‐Ah^14^‐Az_1_ were assigned, and their chemical shift variations were compared with those of ChTx‐Ah^14^ (Table S1). Small chemical shift variations (from 0.07 to 0.22 ppm) were measured for residues Q^18^, S^24^, R^25^, G^26^, K^27,^ and S^37^ which are located on the same face of the peptide (Figure 5a). Moreover, on the 1D ^1^H NMR spectrum, chemical shift variations of the aromatic protons of Y^36^ were observed. Such chemical shift variations indicate modifications in the chemical environment by the cycloaddition of the Az_1_ ligand on this face in its *trans* state. Noteworthy, R^25^ and K^27^ also belong to the pharmacophore (Figure 3a), so we suspect that the cycloaddition of Az_1_ onto ChTx‐Ah^14^ should also mildly affect the potency of the peptide for K_v_1.2 in the *trans* configuration of the monomer. To follow the structural consequences of the isomerization of *trans* to *cis* for ChTx‐Ah^14^‐Az_1_, we measured quantifiable parameters within the 1D ^1^H spectrum of ChTx‐Ah^14^‐Az_1_ in conditions where a fraction of the peptide would isomerize. The two easiest residues that could be followed by clear chemical shift variations on the 1D ^1^H NMR spectrum were the signals of the two aromatic residues, namely Y^36^ (that belongs to the pharmacophore and is also modulated by cycloaddition of Az_1_) and F^2^ (located on the opposite face and whose position is not modified by the cycloaddition of Az_1_). The reference 1D ^1^H spectrum of ChTx‐Ah^14^‐Az_1_ (at t = 0 min) shows that approximately none of the ligands adopts the *cis* conformation before illumination (Figure 5b). As shown, 365 nm‐illumination of ChTx‐Ah^14^‐Az_1_ to favor the *cis* conformation triggers a distinctive uprising of the δ‐Y^36^ and the ε‐Y^36^ signals while leaving untouched all signals related to F^2^ (Figure 5b). After 500 s of illumination, 24% ChTx‐Ah^14^‐Az_1_ is in the *cis* conformation. The process is reversible (back‐switching to *trans*) upon illumination at 435 nm for 120 s. In contrast, coherent with Figure 4c, almost no thermal relaxation could be observed if the peptide was maintained for 24 h in the dark (Figure S11). With these observations in hand, we propose a model of the position of Az_1_ at the surface of ChTx‐Ah^14^ when switching from *trans* to *cis* configuration. We suggest that most of Az_1_ aligns itself along a cleft surrounded by residues R^25^ and K^27^ and positions its free end in the vicinity of Y^36^ (Figure 5c). The combined repositioning of pharmacophore residues upon cycloaddition of Az_1_ onto ChTx‐Ah^14^ and the further structural changes occurring in the pharmacophoric Y^36^ upon *trans*‐to‐*cis* switching leads to expectations that marked changes in pharmacology should be observed. Noteworthy, the 24% signal observed here for the *cis* isomer differs from the 39.2% observed in Figure 4d. One possibility is that different orientations of Az_1_ at the surface of ChTx may coexist, with a fraction of them not affecting Y^36^. Alternatively, differences in pH, buffer, and concentrations used for these experiments may affect these proportions.

### ChTx Potency Modulation by Az_1_ Isomerization

**Figure 6:**
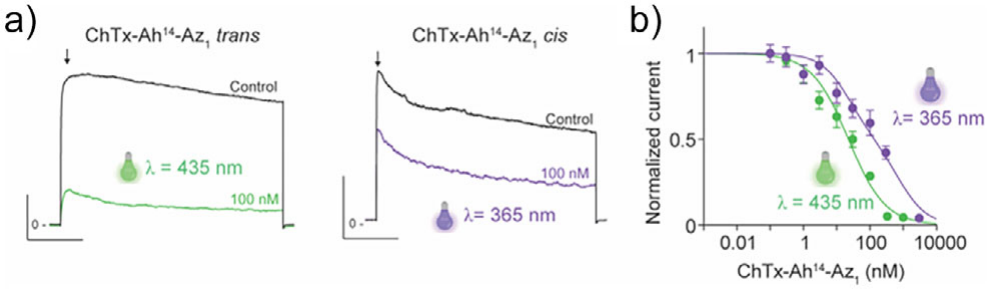
Functional impact of Az_1_ isomerization at the surface of the ChTx‐Ah‐Az_1_ monomers on K_v_1.2 currents. Purple traces are for *trans* conformation and green for *cis*. a) Comparison of K_v_1.2 current block by 100 nm ChTx‐Ah^14^‐Az_1_ in *trans*‐state vs *cis*‐favored state. Peak currents are measured by convention. b) Concentration‐response curves for ChTx‐Ah^14^‐Az_1_ in the two isomerization states. For ChTx‐Ah^14^‐Az_1_ in *cis*‐favored configuration, two IC_50_ values are measured with 22.5 nm (n = 64; 49% *trans*) and 757 nm (n = 64, 51% *cis*). In all‐*trans* configuration, the IC_50_ = 22.5 nm (n = 78). For these experiments, illumination at 365 or 435 nm was maintained before and during the application of the peptides to minimize the impact of channel interaction on unwanted isomerization.

While ChTx‐Ah^14^ had little impact on the potency of ChTx (Figure S5), as expected by a mutation positioned outside the pharmacophore of ChTx, a greater impact of the cycloaddition of Az_1_ to ChTx‐Ah^14^ is expected because of steric hindrance. Indeed, 10 nm ChTx or ChTx‐Ah^14^ block equivalent amounts of K_v_1.2 current (62.2 ± 3.0%, n = 11 vs 52 ± 3%, n = 18), while ChTx‐Ah^14^‐Az_1_ in *trans* blocks a lower amount (37 ± 6%, n = 7) (Figure S12a). Concentration‐response curves confirmed the functional impact of the cycloaddition illustrating that ChTx‐Ah^14^‐Az_1_ is 2.5‐fold less potent than ChTx‐Ah^14^ (IC_50_ values of 9.8 nm without Az_1_ and 22.5 nm with *trans* Az_1_, n = 78) (Figure S12b). Next, the effect of *trans*‐to‐*cis* switching by UV illumination was evaluated on block potency. At 100 nm, the *cis* conformer of ChTx‐Ah^14^‐Az_1_ is 2‐fold less potent than the *trans* conformer (block of K_v_1.2 currents of 36 ± 8% (n = 7) vs 71 ± 2% (n = 11)) (Figure 6 a). This reduction in potency is further supported by the concentration‐response curve of ChTx‐Ah^14^‐Az_1_ in *cis* that was deconvoluted into two components, one coherent with the remaining *trans* fraction (IC_50_ = 22.5 nm, n = 64), and the second to the *cis* one (IC_50_ = 757 nm, n = 64) (Figure 6d). Therefore, the *cis* configuration displays a 34‐fold reduction in the potency of the ChTx‐Ah^14^‐Az_1_ peptide over the *trans* configuration, which is consistent with the structural data illustrating pharmacophore hindrance in *cis* (Figure 5c). It remains a theoretical maximal difference for ChTx‐Ah^14^‐Az_1_ itself since the proportion of *cis* fraction was estimated at 51%. This proportion is higher than the one measured by HPLC (39.2%; Figure 4d) but this technology may potentially underestimate *cis* conformers because of the interactions of the peptide with the column elements.

The functional impact of *trans*‐to‐*cis* isomerization for ChTx‐Ah^9^‐Az_1_, ChTx‐Ah^12^‐Az_1,_ and ChTx‐Ah^19^‐Az_1_ monomers was also assessed (Figure S12*c*). This isomerization had almost no impact on ChTx‐Ah^19^‐Az_1_ potency (IC_50_ = 6.8 nm for *trans* (n = 100 cells) vs 4.2 nm for *cis* (n = 43 cells)), which is coherent with Ah^19^ being positioned far away from the pharmacophore (Figure 2a). ChTx‐Ah^9^‐Az_1_ behaved very much like ChTx‐Ah^14^‐Az_1_ with a 218‐fold reduction in potency for the *cis* isomer (IC_50_ = 1.4 nm (n = 79) for *trans* vs 305 nm (n = 77) for 24% *cis*). *Trans* to *cis* isomerization reactions for ChTx‐Ah^12^‐Az_1_ yielded on the contrary a mild 3.4‐fold gain of function (IC_50_ = 2.7 nm (n = 81) for *trans* vs 0.8 nm (n = 43) for *cis*). These data value at least 3 positions out of 4 tested on ChTx as potential modulators of ChTx activity upon Az_1_‐extension and light‐triggered isomerization. They further inform us that E‐to‐Z isomerization can also lead to the gain of function.

Dimers appeared of lower interest in terms of photomodulation. A mild gain of function effects was observed for the ChTx‐Ah^19^‐Az_1_‐Ah^19^‐ChTx dimer (Figure S13). Whether the channel environment influences their *cis* state (because only one peptide moiety can bind per channel) would be pure speculation at this stage.

### Az_2_ with More Complete Photo‐Switching Properties, Grafted at Position 14 of ChTx, Improves Photo‐Modulation of K_v_1.2 Channels

**Figure 7:**
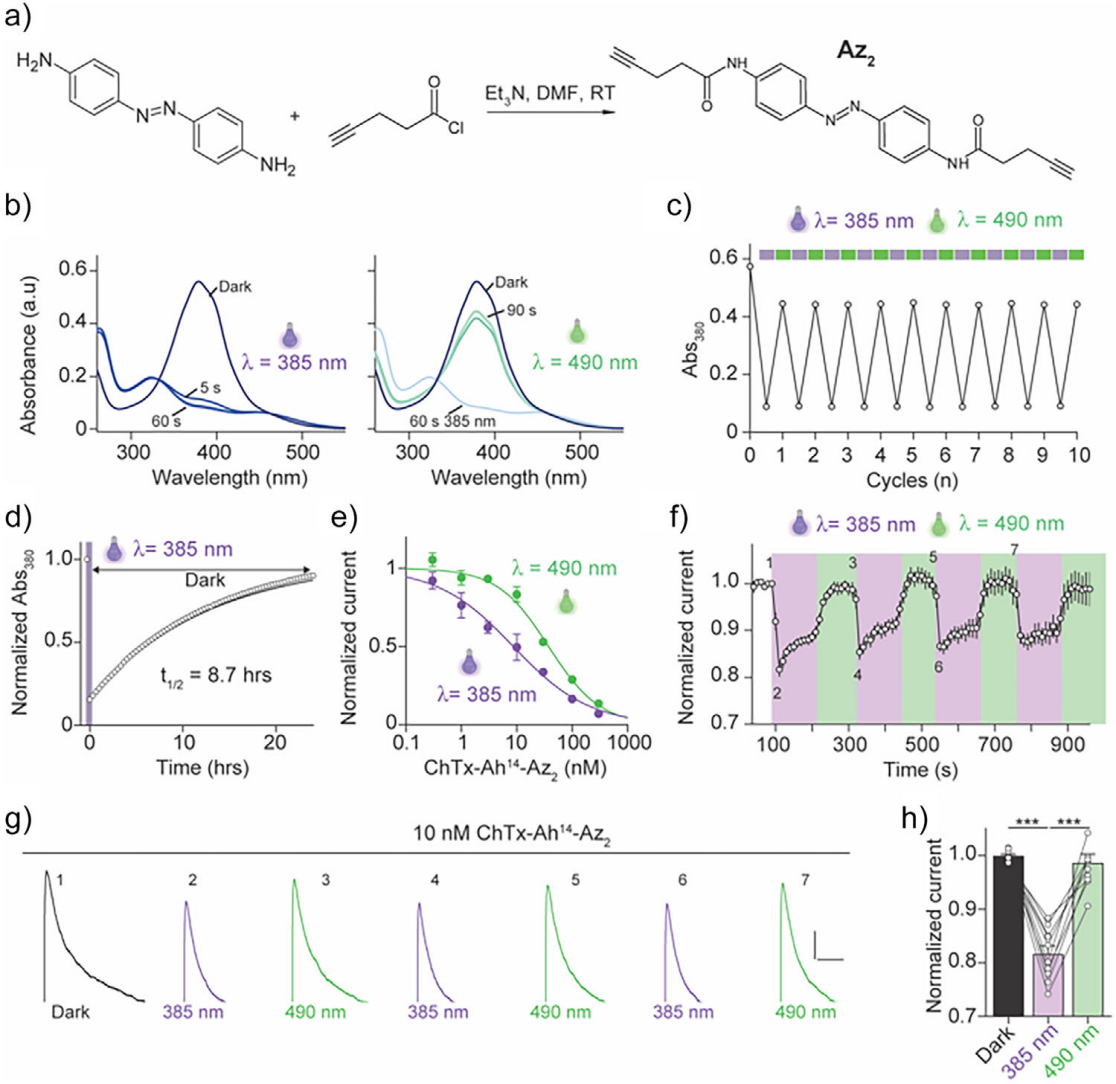
The new Az_2_ photoswitch provides dynamic modulation of K_v_1.2 currents. a) Synthesis pathway of Az_2_. b) Isomerization reaction of Az_2_ promoted by illumination at 9.5 mW cm^−2^ (λ = 385 nm for *trans* to *cis* and λ = 490 nm for *cis* to *trans*) in 100% DMSO. Left panel: traces at 10 and 30 s are superimposed with the trace at 60 s. Right panel: trace at 60 s is superimposed with trace at 90 s. c) Cycles of illuminations to probe the Az_2_ fatigue. d) Stability of the *cis* conformation of Az_2_ over a period of 24 h. e) Concentration‐response curves for ChTx‐Ah^14^‐Az_2_ in the two isomerization states. Measured IC_50_ values are 37.6 nm (n = 41; nH = 0.9; *trans*) and 8.7 nm (n = 40; nH = 0.6; *cis*). f) Average K_v_1.2 current amplitude variation (n = 10) induced by cycles of irradiation at 385 nm (purple) and 490 nm (green). g) Representative K_v_1.2 current traces at 10 nm in the dark and at time points as indicated in (f). Scale bars: 500 ms/1 nA. h) Quantification of the changes in K_v_1.2 current amplitude for the individual cells as a function of wavelength in the presence of 10 nm ChTx‐Ah^14^‐Az_2_ (n = 10 cells studied).

Az_1_‐extension has taught us that monomers behave better than dimers for photo‐modulation of K_v_1.2 and that position 14 is of interest to witness a significant impact of UV illumination on peptide potency. However, since the *trans*‐to‐*cis* isomerization of Az_1_ is incomplete, Az_2_ was synthesized to solve the issue of the extent of E‐to‐Z conversion (Figure 7a). Az_2_ differs from Az_1_ by a more classical amide orientation and by an extended CH_2_ arm on each side. Its complete chemical characterization by ^1^H NMR, ^13^C NMR, and HRMS analyses is provided (Figure S14).

Thermally‐adapted Az_2_ *trans* isomer has a maximal absorption at λ_max_ = 385 nm (Figure 7b). Irradiation at λ = 385 nm (9.5 mW cm^−2^) results in the formation of the *cis* isomer that saturates within 10 s (Figure 7b), which is > 30‐fold faster than Az_1_. Reverse‐switching by 490 nm illumination (9.5 mW cm^−2^) allows for the recovery to *trans* isomer within 60 s, which is a similar time course to Az_1_. However, contrary to Az_1_, the recovery was incomplete and did not reach the dark condition. No significant fatigue was observed after 10 cycles of illumination (Figure 7c). Finally, the thermal relaxation of the *cis* isomer was assessed in dark conditions (Figure 7d). The *cis* isomer of Az_2_ was less stable than Az_1_ (Figure S3) with a half relaxation time of 8.7 h. Next Az_2_ was coupled to ChTx‐Ah^14^ as previously described (Figure S15). Concentration‐response curves of ChTx‐Ah^14^‐Az_2_ were built in both illumination conditions (Figure 7e). In *trans* configuration at 490 nm, K_v_1.2 currents are inhibited by ChTx‐Ah^14^‐Az_2_ with an IC_50_ value of 37.6 nm which is 1.5‐fold less potent than the *trans* configuration of ChTx‐Ah^14^‐Az_1_. This mild potency reduction can be attributed to the longer CH_2_ arms of Az_2_ leading to increased steric hindrance. The dose‐response curve of ChTx‐Ah^14^‐Az_2_ illuminated at 385 nm to favor *cis* configuration shows, contrary to ChTx‐Ah^14^‐Az_1_, a 4.3‐fold increased potency with an IC_50_ value of 8.7 nm. This result indicates that Az_2_ in *cis* does not orient similarly at the surface of ChTx‐Ah^14^ than Az_1_. Here again, this difference in effect may be attributed to the longer arms of Az_2_ and possibly the amide inversion. The Hill slope of the curves also differs (nH = 0.9 in *trans* vs 0.6 in *cis*) suggesting that Az_2_ in its *cis* configuration bends onto the surface of ChTx‐Ah^14^ according to several different schemes, something that was suspected also for the *cis* configuration of ChTx‐Ah^14^‐Az_1_ (Figure 5b). By positioning itself along several configurations over ChTx, Az_2_ in *cis* probably favors a gain of activity only for a fraction of these configurations, explaining why this dose‐response curve spans a large interval of concentrations (from 0.1 nm to 1 µm). One of the hallmarks of photo‐switchable compounds is to provide a dynamic regulation of ion channel activity during the course of the recording (Figure 7f–h). To this end, K_v_1.2 currents were monitored in the presence of dark‐adapted 10 nm ChTx‐Ah^14^‐Az_2_. Illumination of the cells at 385 nm triggered an almost immediate increase in K_v_1.2 current block that reached a peak within 20 s, a value that is fully coherent with the time course required for *trans* to *cis* isomerization of Az_2_ (Figure 7b). Back‐switching of ChTx‐Ah^14^‐Az_2_ by illumination at 490 nm restores initial current levels with a slower kinetics that peaks within ≈50 s, again also coherent with the kinetics of the isomerization reaction of Az_2_ alone. These data indicate that the k_on_ and k_off_ of the toxin onto K_v_1.2 are not rate‐limiting steps. The observed dynamic regulation of K_v_1.2 currents could be reproduced several times. An interesting observation was that back‐switching by 490 nm restores almost the initial dark level of current (Figure 7h) which was not expected from Az_2_ properties alone (Figure 7c). This improved recovery of the *trans* configuration was confirmed by absorbance measurements (Figure S16) indicating that cycloaddition onto the peptide improved the back‐switching amplitude.

Overall, these results indicate that i) the nature of the Az used for extension at position 14 influences the outcome of photoswitching (inhibition or activation) and ii) dynamic regulation of ion channels by complex photoswitchable peptides is feasible.

## Conclusion

Numerous photo‐switching compounds have been developed to regulate ion channel activity in excitable tissues. However, these compounds have primarily been based on small molecules,^[^ ^4^, ^6^, ^13^, ^37^, ^38^, ^39^, ^40^, ^41^, ^42^, ^43^, ^44^ ^]^ sometimes employing a tethering strategy to enhance selectivity for specific ion channels.^[^ ^15^, ^45^, ^46^, ^47^, ^48^ ^]^ Our study represents the first exploration of a photo‐switching strategy utilizing a natural peptide with intricate disulfide bridge patterns. In the case of ChTx, which contains three disulfide bridges, there are potentially 15 combinations of bridging, but only one is active. Thus, it is crucial to maintain proper peptide folding amidst mutagenesis and chemical modifications. We achieved this goal by post‐folding chemical modification of Ah‐substituted ChTx analogs, ensuring both proper folding and preservation of peptide bioactivity. For that purpose, we employed click chemistry conjugation of two new azobenzene compounds with alkyne functions. This post‐folding/oxidation synthesis strategy proved functional across four analogs, indicating robustness and confirming earlier findings describing fluorescent dye^[^ ^31^, ^32^, ^33^ ^]^ or anticancer cargo^[^ ^34^ ^]^ incorporation onto analogous natural peptides. The end result is the successful production of an Az‐extended ChTx that keeps acting on the K_v_1.2 channel at nanomolar concentrations and whose blocking potency undergoes reversible photo‐modulation. However, this finding came at the expense of a learning process that reflects the difficulties in adapting photo‐switches to larger pharmacophores.

Adapting photo‐switches to natural peptides reveals important insights. First, proper positioning of Az for optimal photo‐modulation of peptide efficacy is crucial, with some positions showing greater potency than others. Avoiding the pharmacophore itself is prudent, as Az‐extension directly affects pharmacological potency. However, residues close to the pharmacophore exhibit significant photo‐switching effects. There is thus a tradeoff to respect between largely impacting peptide activity and moderately affecting it by Az addition. Indeed, the cycloaddition of Az at position 14 provided the best results probably because Ah mutation at this particular position was the most detrimental to peptide potency. Azo‐extension of residues located further away from the pharmacophore provided minimal effects, except maybe in the case of ChTx‐Ah^12^‐Az_1_ for which the simple substitution of E^12^ by Ah had an indirect impact for reasons that are unrelated to the pharmacophore (modification of the dipole moment). Second, the kinetics of Az_1_ reverse photo‐switching once grafted onto the peptide indirectly gauged the efficiency of photo‐modulation in our examples. Fast reverse switching appeared to be accompanied by a reduced chance of seeing a difference in peptide potency between *trans* and *cis*. ChTx‐Ah^19^‐Az_1_ has fast reverse switching and also minimal *trans* and *cis* variations in K_v_1.2 block potency. Thirdly, the limited pharmacological impact of ChTx photo‐switching is witnessed for the four dimers, all with fast reverse switching. The effect of channel environment on reverse switching is unknown and one may speculate that it may favor *cis* to *trans* transitions upon peptide binding. This hypothesis may reasonably explain why dimers were less efficient than monomers in creating efficient peptide photo‐switches.

One important information relates to the relative position of Az with regard to peptide structure and how it is impacted upon isomerization. For obvious reasons of workload, it is not possible to investigate the structural consequences of all Az‐cycloadditions isomerization processes. However, investigating the ChTx‐Ah^14^‐Az_1_ structure by NMR taught us interesting information. First, it confirms that azo‐extension at position 14 structurally impacts the pharmacophore when Az_1_ is in *trans* configuration. This effect explains the slight decrease observed in pharmacological potency for this analog on K_v_1.2 and is a positive indication that isomerization will affect potency as well. Second, despite incomplete *trans*‐to‐*cis* isomerization, the chemical environment of Y^36^, an important pharmacophore residue, is modified by isomerization. This observation is also confirmed by the reduced blocking potency of this analog. While structural data provide interesting hints of how potency should evolve during isomerization, it may not be fully predictive of the real situation. We suspect that not all of the *cis*‐favored Az_1_ bends over onto Y^36^, suggesting that alternative less‐favored orientations may occur. This suspicion is reinforced by the fact that substituting Az_1_ with Az_2_, which differs only by the amide orientation and a CH_2_ extension in each arm, results in a gain of function of Az‐extended ChTx‐Ah^14^ rather than a loss of function. Therefore, it is not the nature of the residue on which Az‐extension is performed that matters the most for the structural impact on ChTx, but rather the chemical nature of Az. This is a rather exciting finding since, as soon as a sensitive peptide position is identified for Az‐extension, variations in Az identity will offer a wealth of opportunities to design gain‐or loss‐of‐function effects of isomerization. Noteworthy, the gain of potency obtained with the ChTx‐Ah^14^‐Az_2_ at nanomolar potency in the *cis* configuration is one of the most difficult aims to reach for those who work in the field.

So, what may be the next frontiers to improve natural peptide photo‐switching? Among obvious recommendations, one would be to further chemically modify Az to make use of blue‐ or red‐shifted wavelengths and avoid UVs. Alternative photo‐switches to Az also exist and may potentially be adapted provided that their extension onto the peptide is facilitated. To avoid random Az bending at the surface of the peptide in the *cis* conformation, strategies may be pursued in which the free end of the photo‐switch gets specifically oriented toward defined residues of the peptide by chemical modifications complementary to given peptide residues (for instance a positive charge to complement a negatively charged residue). Finally, this technology awaits the first in vivo use that can finally take advantage of the preserved high potency of the Az‐extended peptide, something hardly achievable with small compounds without tethering on genetically engineered organisms because of low potency.

To summarize, we provide a proof of concept that natural peptide photo‐switches can be designed to modulate ion channels by light. Provided that thousands of natural peptides exist with great potency and selectivity for a large set of ion channels, the photo‐switch technology appears to have a bright future for this class of compounds.

## Conflict of Interests

M. De Waard is a founder and shareholder of Smartox Biotechnology.

## Supporting information



Supporting Information

## Data Availability

The data that support the findings of this study are available in the supplementary material of this article.
