# Supplementary material for: Photoisomerization of Azobenzene‐Extended Charybdotoxin for the Optical Control of Kv1.2 Potassium Channel Activity
Source: Angew Chem Int Ed Engl. 2025 Mar 9;64(19):e202423278. doi: 10.1002/anie.202423278 (PMC12051786; doi:10.1002/anie.202423278)
Supplement: Supplementary file 1 — Supporting Information [file ANIE-64-e202423278-s001.pdf]

Supporting Information

# Photoisomerization of Azobenzene-Extended Charybdotoxin for the Optical Control of $K_V$1.2 Potassium Channel Activity

Yanis Achouba[a], Basile Peres[b], Steven Ascoët[a], Hervé Meudal[c], Cécile Caumes[d], Claude Zoukimian[d], Hugo Millet[a], Maureen Choteau-Bodor[a], Cathy Carvalhosa[e], Mikael Croyal[a], Fella Bouchama[a], Heike Wulff[f], Stéphane Téletchéa[g], Rémy Béroud[d,e], Eléna Ishow[h], Céline Landon[c], Ahcène Boumendjel[i], Jérôme Montnach[a]*, Michel De Waard[a,d,j]*

## Experimental procedures

Chemical synthesis of click chemistry-compatible azobenzene compounds

*$Az_1$* - Triethylamine (0.34 mL, 2.43 mmol) and propargylamine (0.21 mL, 3.26 mmol) were added to a solution of (E) azobenzene-4,4'-dicarboxylic acid dichloride (250 mg, 0.81 mmol – CAS 78752-50-8, Alpha Aesar) in dimethylformamide (20 mL) and the reaction mixture was stirred at room temperature for 24 hrs away from light. Next, 15% aqueous sodium hydroxide (15 mL) was added to the mixture and the reaction was kept under stirring for an additional 24 hrs to hydrolyze the excess of unreacted dichloride derivative. Water was then added to the mixture and the aqueous layer was extracted with ethyl acetate. The organic layer was washed with water, brine and dry over magnesium sulfate before being evaporated under reduced pressure, a process that afforded 70 mg of the desired compound (synthesis yield = 25%), called bis-alkyne-azobenzene (abbreviated $Az_1$ throughout the manuscript).

*$Az_2$* - To a solution of (*E*)-4,4'-(diazene-1,2-diyl)dianiline (106 mg, 0.0005 mmol) and triethylamine (0.3 mL, 2 mmol) in dimethylformamide (6 mL) was added dropwise pent-4-ynoyl chloride (0.21 mL, 2 mmol) and the reaction mixture was kept at room temperature for 3 hrs. Then, the reaction mixture was poured into 60 mL of an aqueous solution of HCl 0.5 N and the formed precipitate was collected by filtration, washed with water, ether and dried under vacuum with a desiccator at room temperature to afford the corresponding amide as a beige solid.

Molecular modelling

To target the most potent amino acids of ChTx peptide for grafting azobenzene without affecting peptide properties, molecular modelling was performed. The structures of $K_V$1.2-2.1 paddle chimera with charybdotoxin (pdb code 4JTA) [1] was used as a template for i) illustrating the docking of ChTx onto $K_V$1.2 and highlighting position of targeted amino-acids and ii) modelling monomers and dimers of ChTx with the azobenzene grafted onto the selected amino-acids. Discovery Studio (Dassault System) was used to build ChTx analogues structures. After applying a fast, Dreiding-like forcefield in order to clean geometry of designed peptide, structures were exported in PyMOL (http://www.pymolwiki.org).

Chemical synthesis of click chemistry-compatible ChTx analogues

For all analogues of ChTx, one amino acid residue was chosen ($T^9$, $E^{12}$, $W^{14}$ or $R^{19}$) to be replaced by L-azidohomoalanine (abbreviated Ah) to render peptides compatible with click chemistry conjugation. Linear ChTx $Ah^9$, ChTx $Ah^{12}$, ChTx $Ah^{14}$ and ChTx $Ah^{19}$ were assembled stepwise using Fmoc SPPS on a

PTI Symphony synthesizer at a 0.1 mmol scale on 2-chlorotrityl chloride resin (substitution approx. 1.6 mmol/g). Fmoc protecting groups were removed using 20% piperidine in DMF and free amine was coupled using tenfold excess of Fmoc amino acids and HCTU/DIEA activation in NMP/DMF (3x15 min). Linear peptides were deprotected and cleaved from the resin with TFA/$H_2O$/1,3-dimethoxybenzene/TIS 92.5/2.5/2.5/2.5 (vol.), then precipitated out in cold diethyl ether, the resulting white solids were washed 2 times with diethyl ether, re-suspended in $H_2O$/acetonitrile and freeze dried to afford crude linear peptide. Next, the crude peptides were folded by air oxidation at 25 mM in a 0.1 M TRIS buffer at pH 8.0, containing 5 mM GSH and 0.5 mM GSSG. After 24 hrs the pH of the reaction mixtures was adjusted to 3 and the peptides were purified by reversed phase HPLC on a C18 Phenomenex Luna stationary phase on an Agilent Technologies preparative HPLC system (eluent system $H_2O$/MeCN + 0.1% TFA). As a result, pure synthetic ChTx-$Ah^{9}$, ChTx-$Ah^{12}$, ChTx-$Ah^{14}$ and ChTx-$Ah^{19}$ were obtained.

Click chemistry coupling of ChTx Ah analogues to Az for the production of azobenzene-coupled peptide monomers and dimers

The ChTx Ah peptide analogues were solubilized by sequential addition of equal volumes of NMP/tBuOH 9:1, distilled water and 0.4 M HEPES containing 50 mM of aminoguanidine to a final peptide concentration of 15 mM. Next, 0.33 (monomer-favored production condition) or 3 equivalents of Az (dimer-favored production condition) were solubilized in a minimum volume of NMP/tBuOH 9:1 and added to the peptide solutions which were degassed and flushed with Ar. Later, 10 equivalents of Copper(I) bromide dimethylsulfide complex and 10 equivalents of THPTA (Tris-(3-hydroxypropyltriazolylmethyl)amine) were dissolved in a minimum volume of NMP/tBuOH 9:1 and added to the peptide Ah / Az solutions. The pH of the reaction mixtures was checked and adjusted to 7-8, if required, before being degassed and flushed with Ar, then allowed to stir overnight at 40°C. The reactions were quenched by acidification with formic acid and the mixtures were immediately diluted with 6 M guanidine hydrochloride before purification of all new conjugates (peptide monomers and dimers) by RP-HPLC. To estimate peptide monomer and dimer weights after syntheses, a correction factor had to be applied because of the absorbance at 280 nm of the Az moiety ($^{280}e=7365\ L.mol^{-1}.cm^{-1}$, E isomer) such that $m = 0.66 \times m_{weight}$. The above correction factor was used similarly for the quantification of the monomers and of the dimers: $R^{19}$ 0.74, $T^{9}$ 0.56, $E^{12}$ 0.66 and $W^{14}$ 0.92.

UV-visible spectrometry

Photoswitching of the Az alone or of each ChTx analogue was quantified thanks to UV-vis absorption spectroscopy (Agilent, Cary 5000 UV-Vis-NIR spectrophotometer) measured from 800 to 200 nm before and after illumination. Photoswitching was induced by using a Xe-Hg source (Hamamatsu, LC8 Lightningcure Spot light source), equipped with a bundle of quartz fibers and narrow bandpass filters (Semrock, Brightline®) centered at 340 nm (FF01-340/12-25) or 435 nm (FF02-435/40-25). Illumination intensity was set at 9.5 mW/cm² using a power meter (Ophir, Nova II) and a PD300 photodiode sensitive to the UV range. Back thermal relaxation was measured at 20°C and acquisition was performed every 20 min after 340 nm illumination. Illumination at 435 nm was performed after 24 hrs to fully restore the E isomer.

NMR spectrometry

Solutions of ChTx-$Ah^{14}$ (dissolved in 220 µL $H_2O/D_2O$ (95/5)) and ChTx-$Ah^{14}$-Az (dissolved in 220 µL $D_2O$) peptides were prepared in 3-mm NMR tubes. All spectra were acquired on a BRUKER 700 MHz NMR

spectrometer equipped with a 5-mm TCI cryoprobe at 298 K. Processing and analyses were performed with Bruker's TopSpin3.2 and CcpNMR softwares. Photoswitching of the azobenzene, induced by using an 8W EB-180C spot light source (Spectroline) at 312 nm, was followed by 1D $^{1}H$ NMR. A series of acquisitions was performed after 3 to 50 min of illumination. An additional spectrum was acquired after 2 days (without illumination) to check the reversibility of the process and the return to the *trans* conformation. Homonuclear TOCSY and NOESY spectra, and $^{13}C$ HSQC (natural abundancy), were acquired to allow the assignment of Hα chemical shifts for both the ChTx-Ah$^{14}$ and the major form of the grafted protein, ChTx-Ah$^{14}$-Az in *trans* conformation. Variations in Hα chemical shifts between the two molecules were calculated. Hα protons chemical shift assignments could not be obtained for ChTx-Ah$^{14}$-Az in *cis* isomer, that reach a low concentration of 50 µM. The Az part of ChTx-Ah$^{14}$-Az was manually oriented in both *cis* and *trans* ChTx analogue structures to reflect the NMR data (chemical variations for Hα and aromatic protons), and minimized with Chimera1.17.3 software[2], without constraints, to avoid steric clashes.

Monitoring of Photostationnary states (PSS) of ChTx analogues

Analytical RP-HPLC was performed using an SPD M20-A system (Shimadzu) with a Luna OmegaPS C18 column (4.6 x 250 mm, 5 µm, 100 Å). 20 µL (corresponding to 7 µg of material) was loaded and a 5-60% acetonitrile gradient (0.1% TFA v/v) was applied over 35 min (except for ChTx-Ah$^{14}$-Az-Ah$^{14}$-ChTx dimer) at room temperature to detect analytes by UV absorbance at 214 nm. Illumination of samples was performed at 365 nm for different times (between 1 sec and 30 min illumination time) at 41.8 mW/cm$^2$ or less (as specified in the *Result section*) for 10 min using a CoolLED pE4000 light source (CoolLED, UK). Flash energy has been measured using a highly sensitive thermal power head (S401C, ThorLabs).

Cell culture

L929 cell line stably expressing murine (m)$K_v$1.2 were cultured Dulbecco's Modified Eagle's Medium (DMEM) supplemented with 10% fetal bovine serum, 1 mM pyruvic acid, 4.5 g/L glucose, 4 mM glutamine, 800 µg/mL G418, 10 U/mL penicillin and 10 µg/mL streptomycin (Gibco, Grand Island, NY). Cells were incubated at 37°C in 5% $CO_2$ atmosphere.

Automated patch clamp recordings

The ChTx analogues were investigated on L929 cells expressing m$K_v$1.2 channel using an automated patch-clamp system (SyncroPatch 384PE from Nanion, München, Germany). Cells were isolated with trypsin and diluted in an extracellular solution containing (in mM): 140 NaCl, 4 KCl, 2 $CaCl_2$, 1 $MgCl_2$, 5 glucose and 10 HEPES (pH 7.4, osmolarity 312 mOsm) at a density > 300,000 cells/mL. The intracellular solution contained (in mM): 110 CsF, 10 CsCl, 10 NaCl, 10 EGTA and 10 HEPES (pH 7.2, osmolarity 260 mOsm). The PatchControl384 v1.5.2 software (Nanion) was used for application of voltage pulses and whole-cell recordings, whereas the Biomek v1.0 interface (Beckman Coulter) was needed for ChTx applications in the extracellular medium. Whole-cell experiments were performed at room temperature (23°C), holding potential was set at -100 mV, and sampling rate was set at 20 kHz for current recordings. Each ChTx analogue was prepared at various concentrations in the extracellular solution, itself supplemented with 0.3% bovine serum albumin (BSA). Compound solutions were diluted 3 times in the patch-clamp recording well by adding 30 µL external solution, to reach the final reported concentration and the test volume of 90 µL. For establishing concentration-response curves, the compounds were tested at a test potential of +10 mV for 50-ms with a pulse every 5 sec. Data were

extracted from PatchControl384 and analyzed in ad hoc R routines to quantify maximal current amplitude. Percentages of current inhibition were measured at steady-state of effect at the end of a 15-min application time.

<u>Statistics</u>

Values are represented as mean ± SEM.

**Figures S1 to S9**

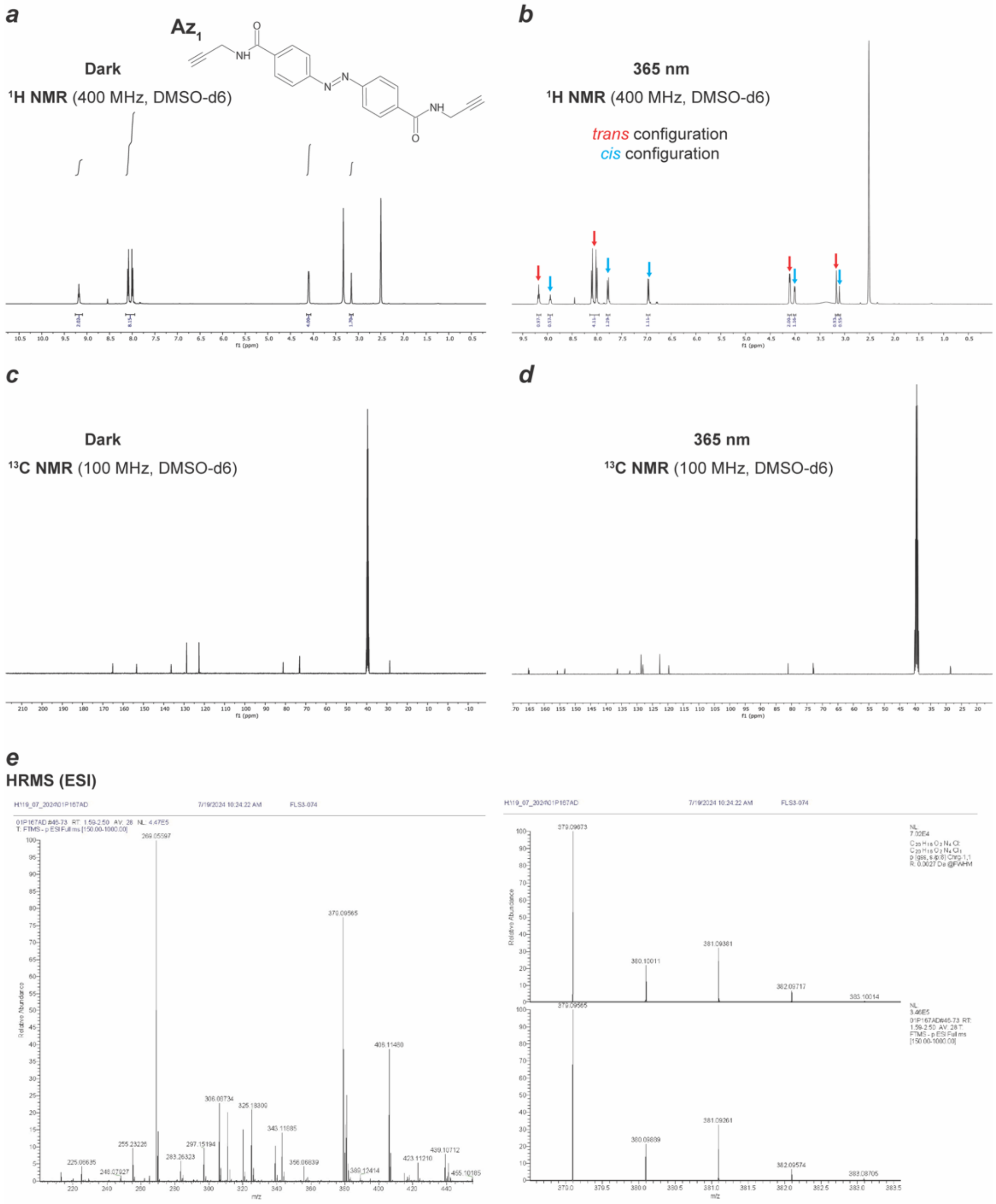


**Figure S1**. **NMR spectra of $Az_1$ compound**. ***a***, **$^1$H NMR** of $Az_1$ in *trans* configuration (400 MHz, DMSO-*$d_6$*) $\delta$ (ppm) 3,15 (t, $J$ = 2.5 Hz, **2H**), 4.10 (dd, $J$ = 5.5 Hz, $J$ = 2.5 Hz, **4H**), 8.00 (d, $J$ = 8.7 Hz, **4H**), 8.09 (d, $J$ = 8,7 Hz, **4H**), 9.18 (t, $J$ = 5.5 Hz, **2H**). ***b***, **$^1$H NMR** of $Az_1$ in mixed *trans* / *cis* configuration. Arrows indicate signals belonging to *cis*. ***c,*** **$^{13}$C NMR** of $Az_1$ in *trans* configuration (100 MHz, DMSO-*$d_6$*) $\delta$ (ppm) 28.6 (**$CH_2$**), 73.0 (**CH**), 81.1 (**C**), 122.6 (**CH**), 128.7 (**CH**), 136.3 (**C**), 153.3 (**C**), 165.1 (**C**). ***d***, **$^{13}$C NMR** of $Az_1$ in

mixed *trans* / *cis* configuration. ***e***, **HRMS (ESI)** *m/z* calc. for $C_{20}H_{16}N_4O_2Cl$ $[M+Cl]^-$ 379.09673, found 379.09565.

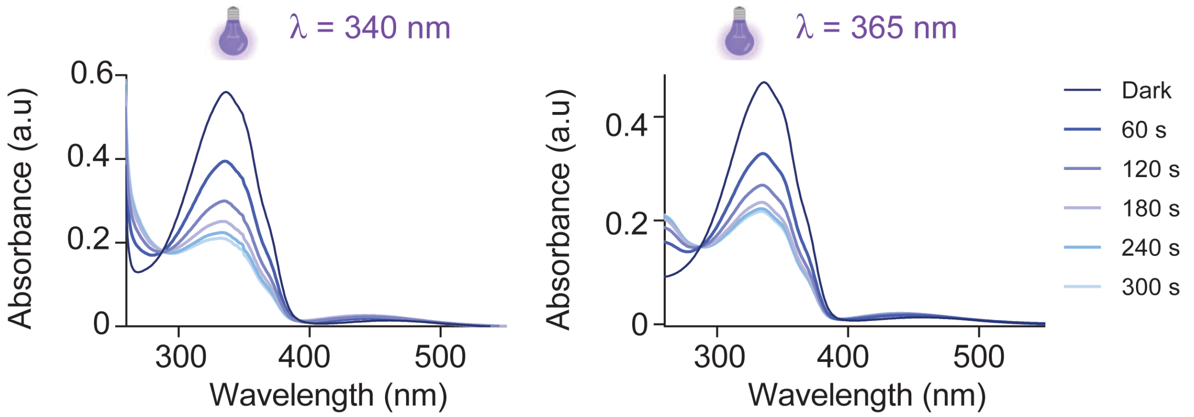


**Figure S2. Isomerization reaction of $Az_1$.** Isomerization reaction of $Az_1$ from *trans* to *cis* was promoted by UV illumination at 340 nm or 365 nm with comparable results.

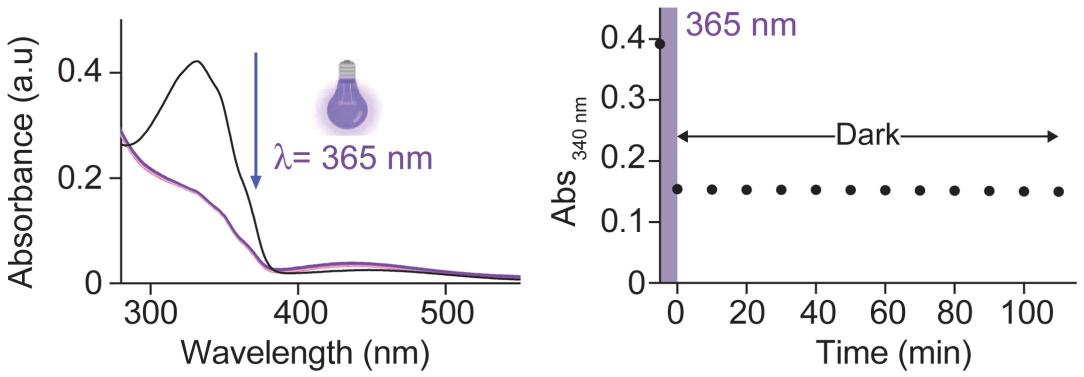


**Figure S3. Thermal relaxation of $Az_1$.** Absorbance spectra of $Az_1$ shifting from dark to *cis* (left panel) and time-dependent evolution of this *cis* isomer maintained in dark over 110 min.

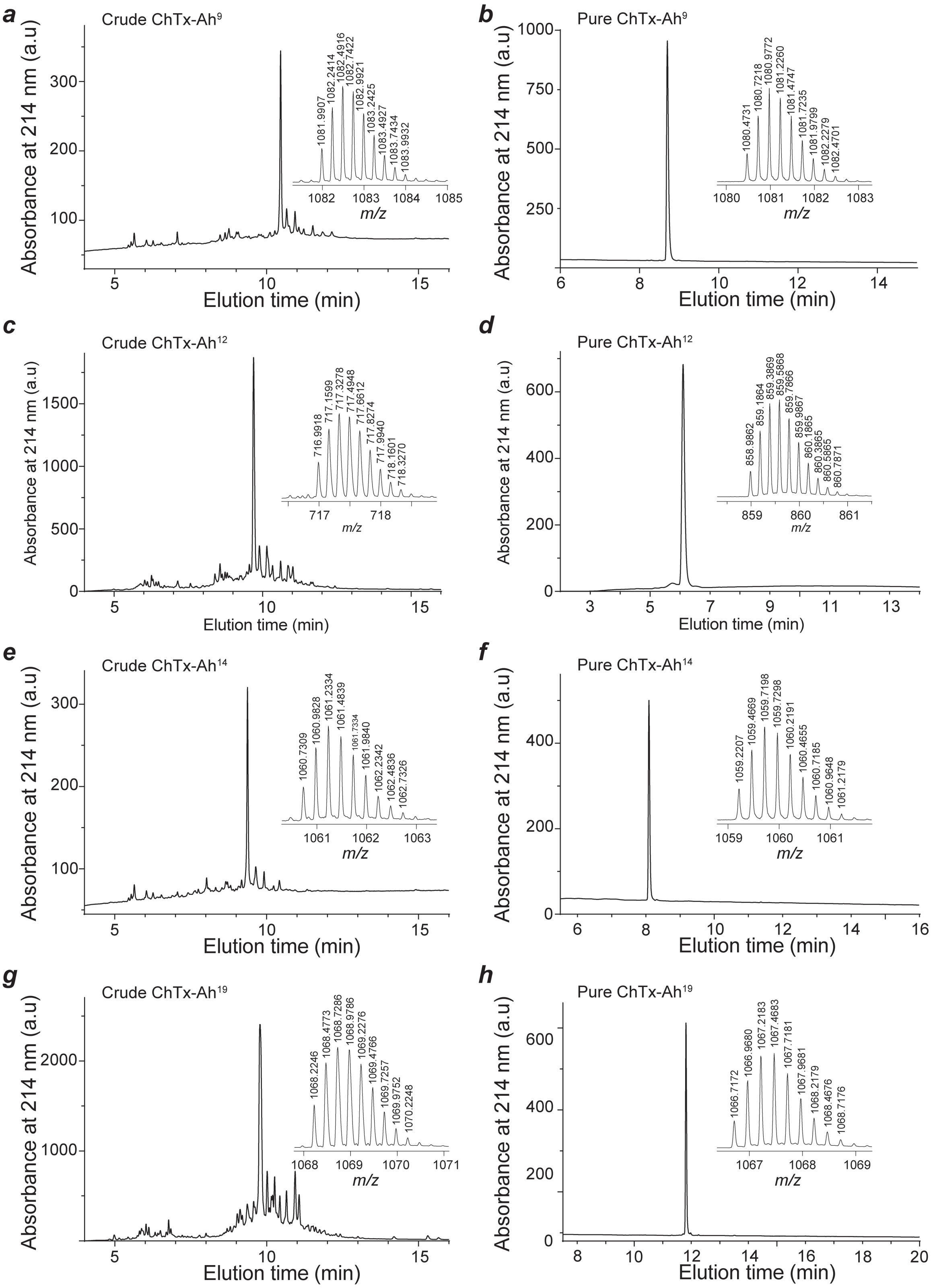


**Figure S4**. **Chemical syntheses and mass spectrometry of click-compatible ChTx-Ah-mutated analogues.** ***a.*** Chemical synthesis of ChTx-Ah$^{9}$. Left panel. crude peptide and [M + 4H]$^{4+}$ mass spectrum. as determined by LC-ESI QTOF. in inset. Right panel. similar representation but for the folded/oxidized and purified ChTx-Ah$^{9}$ peptide. ***b***. ***c*** & ***d***. as in ***a*** but for ChTx-Ah$^{12}$. ChTx-Ah$^{14}$ and ChTx-Ah$^{19}$. respectively. Mass spectra shown are [M + 6H]$^{6+}$ for crude ChTx-Ah$^{12}$. [M + 5H]$^{5+}$ for purified ChTx-Ah$^{12}$ and [M + 4H]$^{4+}$ for the remaining ones.

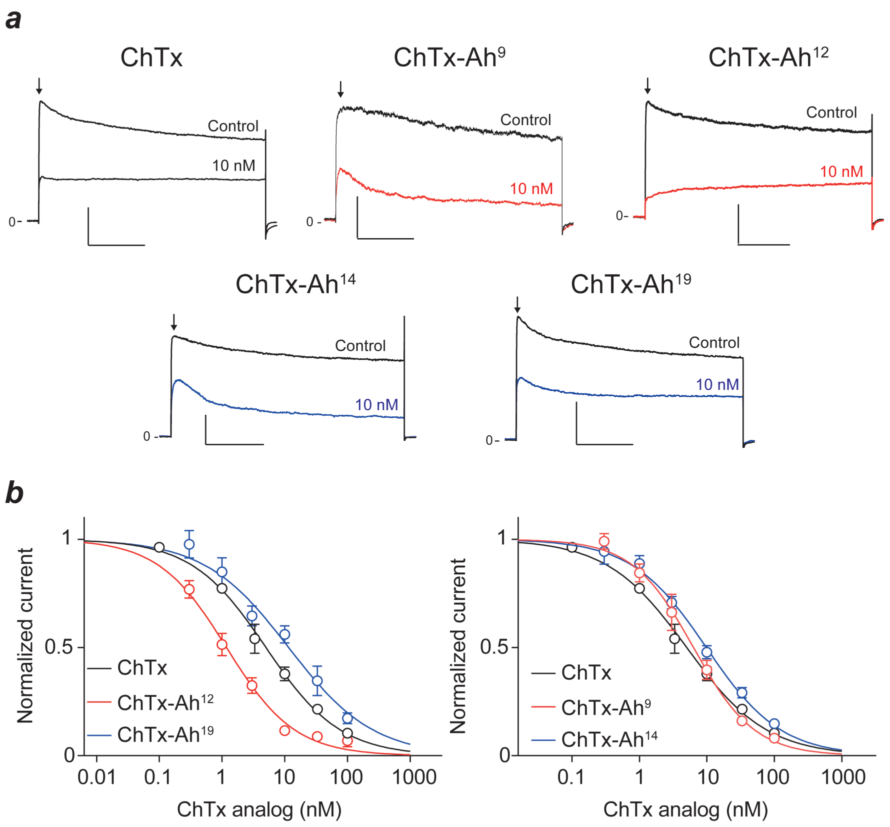


**Figure S5. Impact of ChTx amino acid substitutions by Ah on $K_v$1.2 blocking potency**. ***a***, Representative current traces of $K_v$1.2 currents before and after application of 10 nM ChTx, ChTx-$Ah^9$, ChTx-$Ah^{12}$, ChTx-$Ah^{19}$ and ChTx-$Ah^{14}$. Arrows represent peak level currents that were measured for dose-response curves. ***b***, Concentration response curves illustrating the blocking potencies of the Ah-substituted ChTx analogues compared to wild-type ChTx. Fit of the data by a Hill equation provides the following $IC_{50}$ values and Hill numbers: 5.0 ± 1.1 nM and $n_H$= -0.7 (ChTx, n=59 cells), 1.2 ± 1.1 nM and $n_H$= -0.8 (ChTx-$Ah^{12}$, n=91 cells), 11.5 ± 1.2 nM and $n_H$= -0.6 (ChTx-$Ah^{19}$, n=104 cells), 6.3 ± 1.1 nM and $n_H$= -0.9 (ChTx-$Ah^9$, n=69 cells) and 9.8 ± 1.1 nM and $n_H$= -0.8 (ChTx-$Ah^{14}$, n=76 cells). The gain of potency for the ChTx-$Ah^{12}$ variant can be explained by an improved dipole moment of the peptide, as observed on other occasions upon mutation of a negatively charged residue.[3]

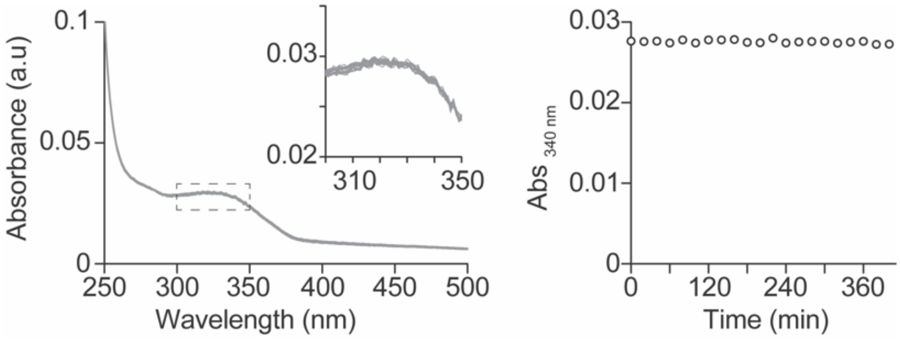


**Figure S6. Stability of ChTx-$Ah^{14}$-$Az_1$ in the presence of 10 mM GSH.** Absorbance spectra of the peptide upon prolonged exposure to 10 mM GSH (> 6 hours), measured every 30 min. Inset: zoom on the 300-350 nm wavelength window. The absorbance value is measured at 340 nm (right panel).

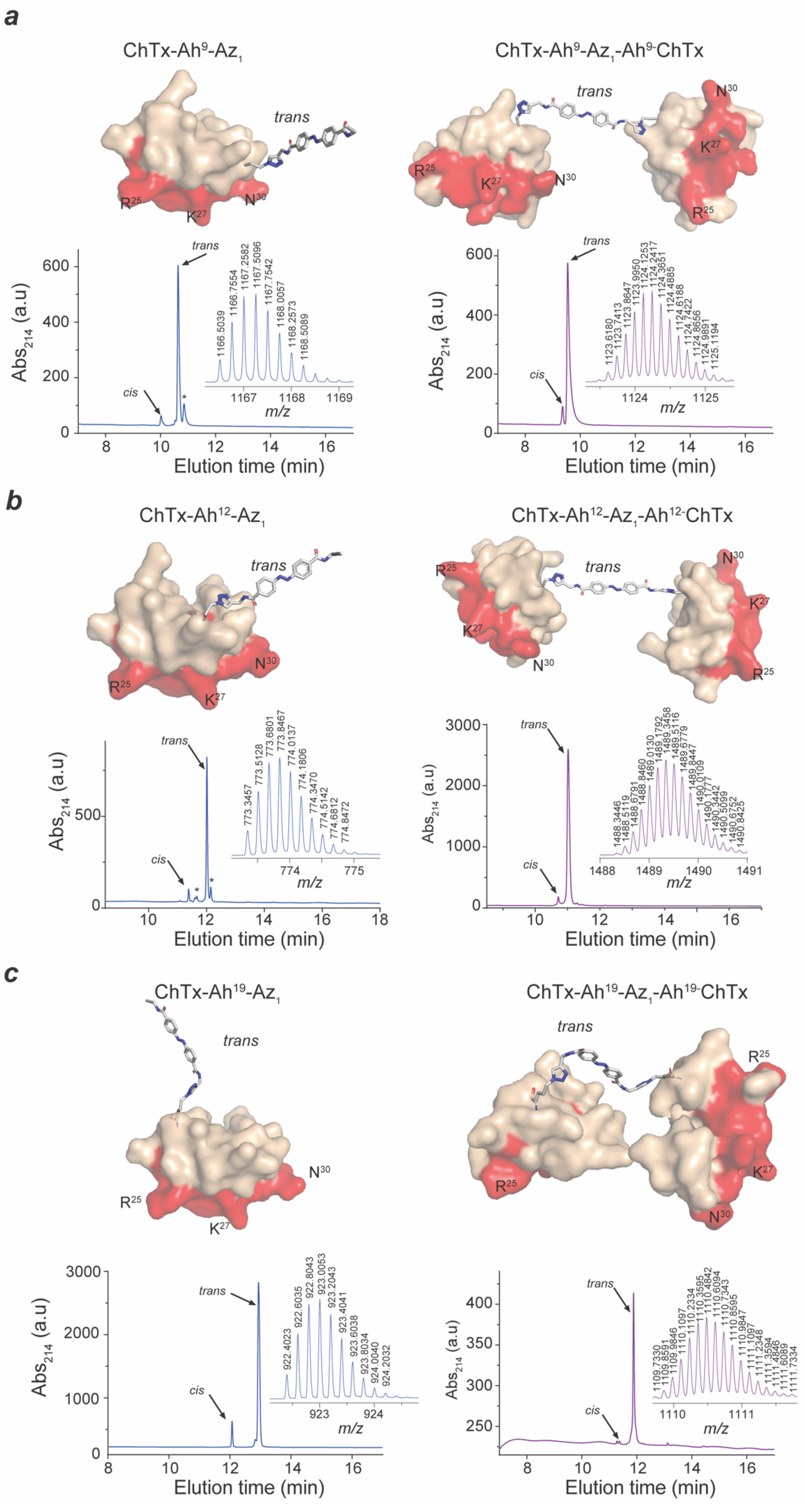
a
ChTx-Ah9-Az1
ChTx-Ah9-Az1-Ah9-ChTx
trans
R25
K27
N30
Abs214 (a.u)
Elution time (min)
m/z
cis
trans
b
ChTx-Ah12-Az1
ChTx-Ah12-Az1-Ah12-ChTx
c
ChTx-Ah19-Az1
ChTx-Ah19-Az1-Ah19-ChTx

**Figure S7. Production and purification of the ChTx-Ah$^{9}$-Az$_1$, ChTx-Ah$^{12}$-Az$_1$ and ChTx-Ah$^{19}$-Az$_1$ monomers and the ChTx-Ah$^{9}$-Az$_1$-Ah$^{9}$-ChTx, ChTx-Ah$^{12}$-Az$_1$-Ah$^{12}$-ChTx and ChTx-Ah$^{19}$-Az$_1$-Ah$^{19}$-ChTx dimers**. ***a***. lower left panel: elution profile and $[M + 4H]^{4+}$ MS (inset) of ChTx-Ah$^{9}$-Az$_1$ monomer after click chemistry conjugation in the 0.33 Az$_1$/1 ChTx-Ah$^{9}$ stochiometric ratio (left panel). 5% of the *cis* conformer is also visible (95% *trans*). Lower right panel: elution profile and $[M + 8H]^{8+}$ MS of ChTx-Ah$^{9}$-Az$_1$-Ah$^{9}$-ChTx dimer after click chemistry conjugation in the 3 Az$_1$/1 ChTx-Ah$^{9}$ stochiometric ratio. 6% *cis* configuration present. ***b***. left panel: elution profile and $[M + 6H]^{6+}$ MS (inset) of ChTx-Ah$^{12}$-Az$_1$ monomer after click chemistry conjugation in the 0.33 Az/1 ChTx-Ah$^{12}$ stochiometric ratio (left panel). 8% of the *cis* conformer is also visible 92% *trans* configuration). Other minor peaks are remaining contaminants. Right panel: Elution profile and $[M + 6H]^{6+}$ MS of ChTx-Ah$^{12}$-Az$_1$-Ah$^{12}$-ChTx dimer after click chemistry conjugation in the 3 Az$_1$/1 ChTx-Ah$^{12}$ stochiometric ratio. 4% *cis* configuration present (96% *trans* configuration) ***c***. left panel: Elution profile and $[M + 5H]^{5+}$ MS (inset) of ChTx-Ah$^{19}$-Az$_1$ monomer after click chemistry conjugation in the 0.33 Az$_1$/1 ChTx-Ah$^{19}$ stochiometric ratio (left panel). 9% of the *cis* conformer is also visible (91% of *trans*). Right panel: Elution profile and $[M + 8H]^{8+}$ MS of ChTx-Ah$^{19}$-Az$_1$-Ah$^{19}$-ChTx dimer after click chemistry conjugation in the 3 Az$_1$/1 ChTx-Ah$^{19}$ stochiometric ratio. *cis* conformer difficult to quantify. Top panels for ***a***. ***b*** and ***c***. schematic 3D representations of the monomers and dimers illustrating the size and length of the Az$_1$ linker in the *trans* configuration. The pharmacophore is in red with K$^{27}$ being the residue entering into K$_v$1.2 channel pore.

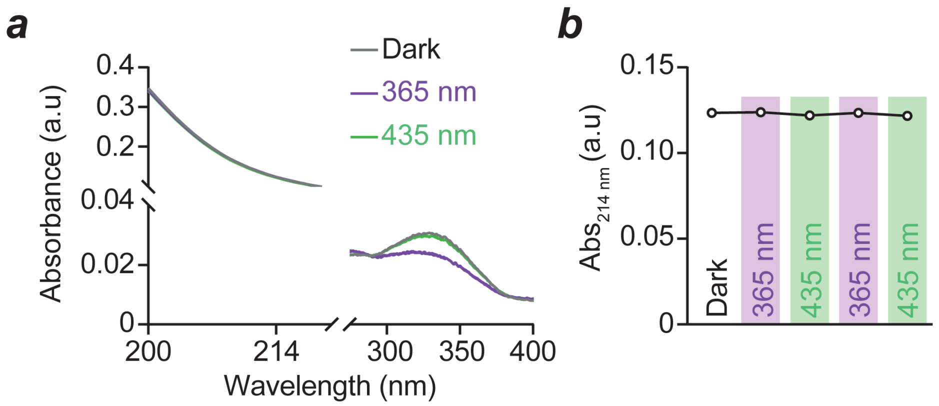


**Figure S8. Absorbance of ChTx-Ah$^{14}$-Az$_1$ at 214 nm is not influenced by *cis* / *trans* isomerization of Az$_1$.** ***a***, comparison of the absorbance spectra of ChTx-Ah$^{14}$-Az$_1$ after illumination at 365 or 435 nm. ***b***, Repeated illumination at 365 or 435 nm does not alter the absorbance value at 214 nm.

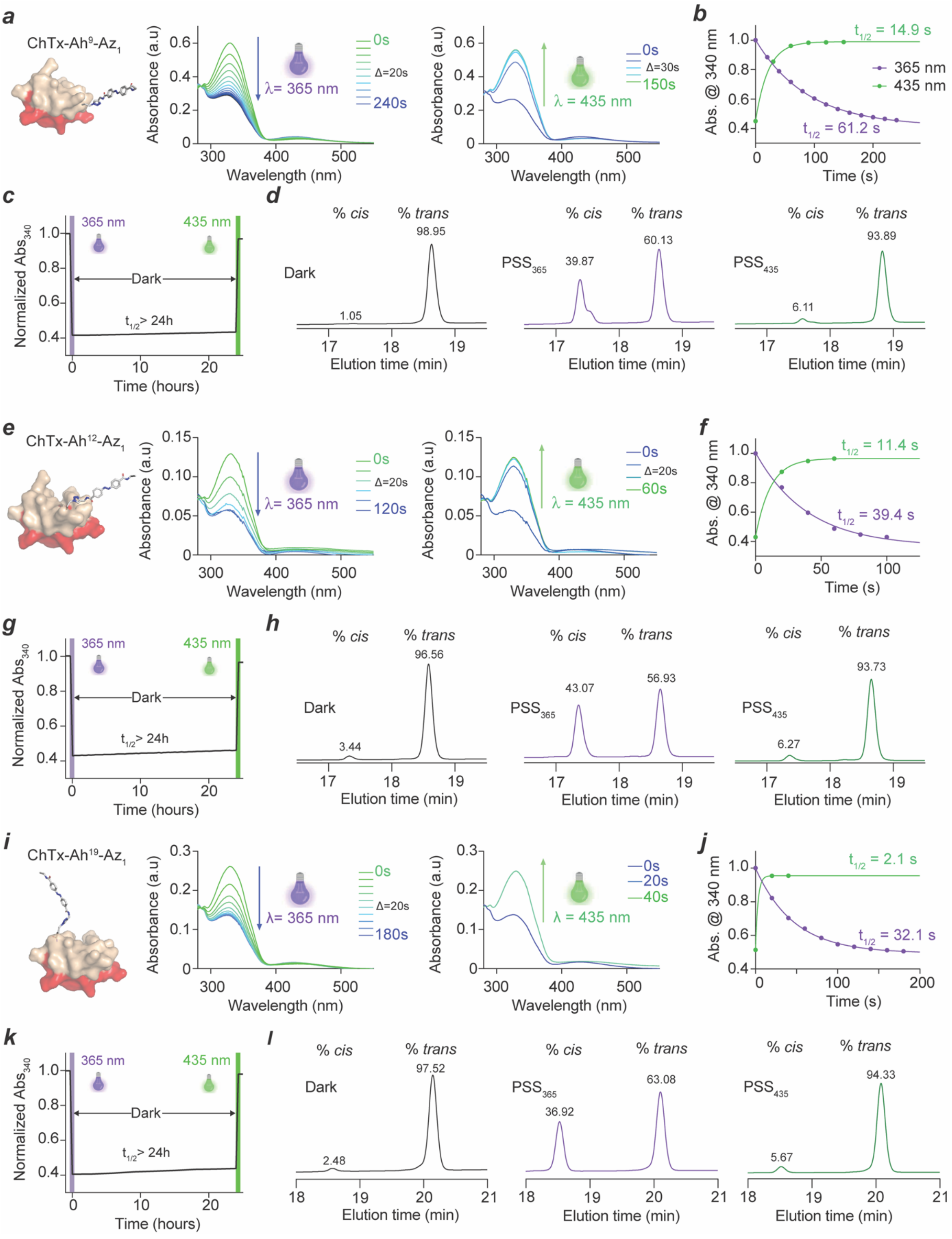


**Figure S9. Photo-isomerization reaction analyses of the three other monomers**. ChTx-Ah$^{9}$-Az$_{1}$. ChTx-Ah$^{12}$-Az$_{1}$ and ChTx-Ah$^{19}$-Az$_{1}$. ***a***. Spectral evolution of the ChTx-Ah$^{9}$-Az$_{1}$ monomer upon irradiation at 340 and 435 nm (9.5 mW.cm$^{-2}$). The monomer is schematized on the left panel. ***b***. Fits of the isomerization data in order to extract the isomerization half-lives ($t_{1/2}$). ***c***. Thermal stability at 20°C of the *cis* isomer of the ChTx-Ah$^{9}$-Az$_{1}$ monomer over a period of 24 hrs. ***d***. HPLC analyses of ChTx-Ah$^{9}$-Az$_{1}$ before illumination (initial state), and in the photo-stationary state (PSS) after illumination at 365 nm (PSS$_{365}$) and upon reverse switching at 435 nm (PSS$_{435}$). ***e-h***. as for ***a-d***. but for the ChTx-Ah$^{12}$-Az$_{1}$ monomer. ***i-l***. as for ***a-d***. but for the ChTx-Ah$^{19}$-Az$_{1}$ monomer.

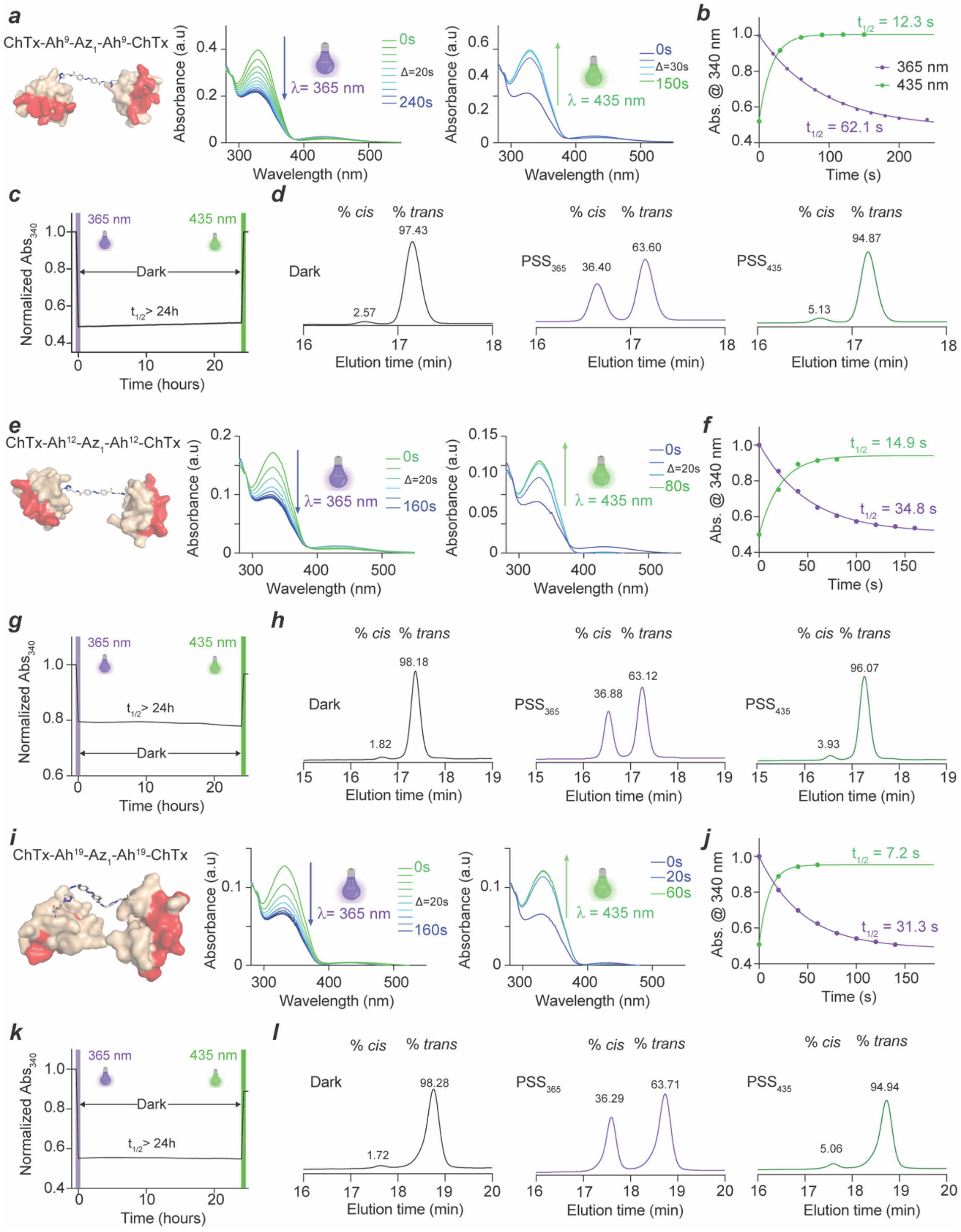


**Figure S10. Photo-isomerization properties of the three other dimers.** ChTx-Ah$^9$-Az$_1$-Ah$^9$-ChTx. ChTx-Ah$^{12}$-Az$_1$-Ah$^{12}$-ChTx and ChTx-Ah$^{19}$-Az$_1$-Ah$^{19}$-ChTx. ***a***. kinetics of isomerization of the ChTx-Ah$^9$-Az$_1$-Ah$^9$-ChTx dimer. schematized on the left panel. ***b***. Fits of the isomerization data in order to extract half-isomerization times ($t_{1/2}$). ***c***. Stability of the *cis* conformation of the ChTx-Ah$^9$-Az$_1$-Ah$^9$-ChTx monomer over a period of 24 hrs. ***d***. Photostationary states of ChTx-Ah$^9$-Az$_1$-Ah$^9$-ChTx in dark. after illumination at 365 nm and upon back-switching by illumination at 435 nm. as assessed by HPLC. ***e-h***. as for ***a-d***. but for the ChTx-Ah$^{12}$-Az$_1$-Ah$^{12}$-ChTx dimer. ***i-l***. as for ***a-d***. but for the ChTx-Ah$^{19}$-Az$_1$-Ah$^{19}$-ChTx dimer.

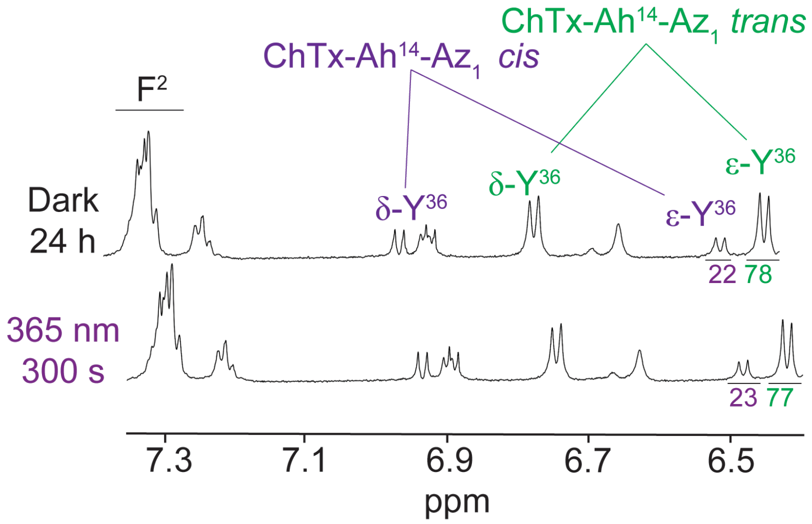


**Figure S11. Thermal relaxation of ChTx-Ah$^{14}$-Az$_1$ over 24 hrs as analyzed by 1D $^1$H NMR**. Conditions as in **Figure 5**.

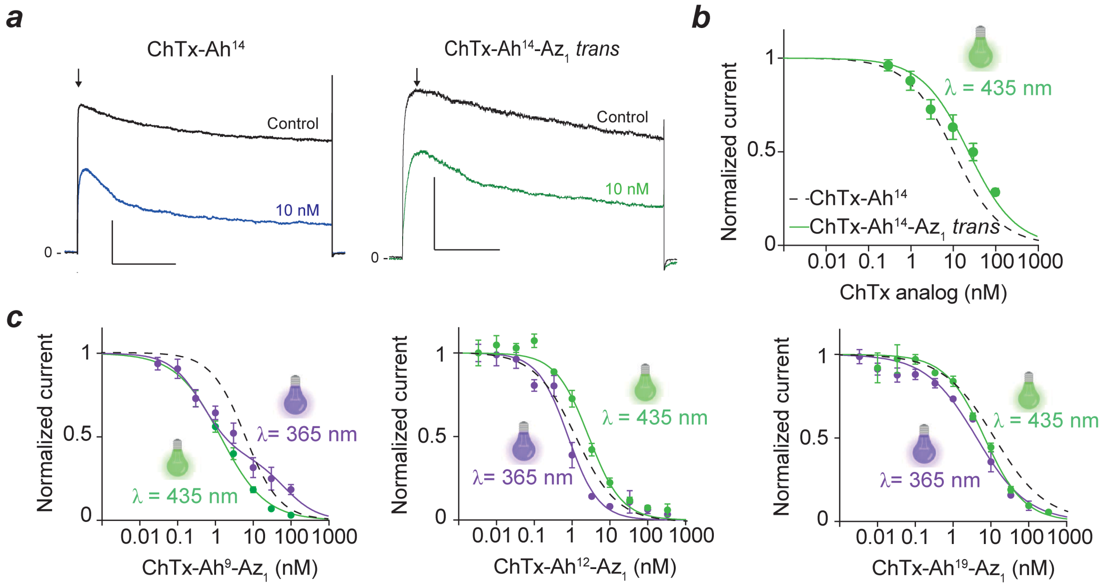


**Figure S12. Impact of Az$_1$ extension on ChTx-Ah$^{14}$ blocking potency of K$_v$1.2 currents**. ***a***, Effects of Ah mutation at position 14 of ChTx and of Az$_1$ cycloaddition onto ChTx-Ah$^{14}$ on K$_v$1.2 current traces at 10 nM concentration. ***b***, Concentration-response curves for ChTx-Ah$^{14}$ and ChTx-Ah$^{14}$-Az$_1$ in the *trans* isomerization state. Data fit yield $IC_{50}$ = 9.8 nM (n=76) for ChTx-Ah$^{14}$ and $IC_{50}$ = 24.5 nM (n=59) for ChTx-Ah$^{14}$-Az$_1$ in *trans*. ***c***, Impact of Az cycloaddition to other mono-substituted ChTx-Ah analogues and of the *trans* to *cis* isomerization on dose-response curves for block of K$_v$1.2 currents. The dotted lines are for Ah-substituted ChTx analogues as provided in ***Figure S5.*** $IC_{50}$ values were as follows. ChTx-Ah$^{19}$-Az$_1$ *trans*: 6.8 nM (n=100); ChTx-Ah$^{19}$-Az$_1$ *cis*-favored: 4.2 nM (n=43). ChTx-Ah$^{12}$-Az$_1$ *trans*: 2.7 nM (N=81); ChTx-Ah$^{12}$-Az$_1$ *cis*-favored: 0.8 nM (n=43). ChTx-Ah$^{9}$-Az$_1$ *trans*: 1.4 (n=79); ChTx-Ah$^{9}$-Az$_1$ *cis*-favored: 1.4 nM (n=77; 76% *trans* component) and 305 nM (n=77; 24% *cis* component).

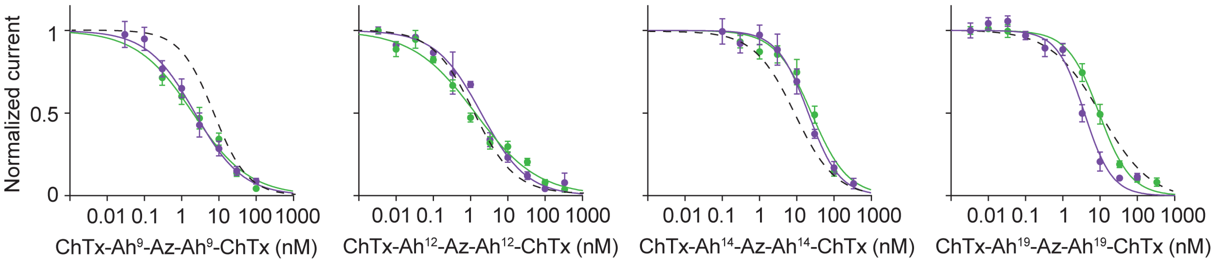


**Figure S13. Dose-response curves for the four ChTx dimers on $K_v$1.2 currents in *trans* and *cis* configurations.** Blue: 365 nm; green: 435 nm. $IC_{50}$ values were as follows. ChTx-Ah$^9$-Az$_1$-ChTx-Ah$^9$ *trans*: 2.0 nM (n=80 cells); ChTx-Ah$^9$-Az$_1$-ChTx-Ah$^9$ *cis*-favored: 2.3 nM (n=104 cells). ChTx-Ah$^{12}$-Az$_1$-ChTx-Ah$^{12}$ *trans*: 1.3 nM (n=91 cells); ChTx-Ah$^{12}$-Az$_1$-ChTx-Ah$^{12}$ *cis*-favored: 1.9 nM (n=33 cells). ChTx-Ah$^{14}$-Az$_1$-ChTx-Ah$^{14}$ *trans*: 24.7 nM (n=66 cells); ChTx-Ah$^{14}$-Az$_1$-ChTx-Ah$^{14}$ *cis*-favored: 20.3 nM (n=80 cells). ChTx-Ah$^{19}$-Az$_1$-ChTx-Ah$^{19}$ *trans*: 9.4 nM (n=89 cells); ChTx-Ah$^{19}$-Az$_1$-ChTx-Ah$^{19}$ *cis*-favored: 3.8 nM (n=38 cells). Dashed curves correspond to the corresponding ChTx-Ah peptides as reference (**Figure S5**).

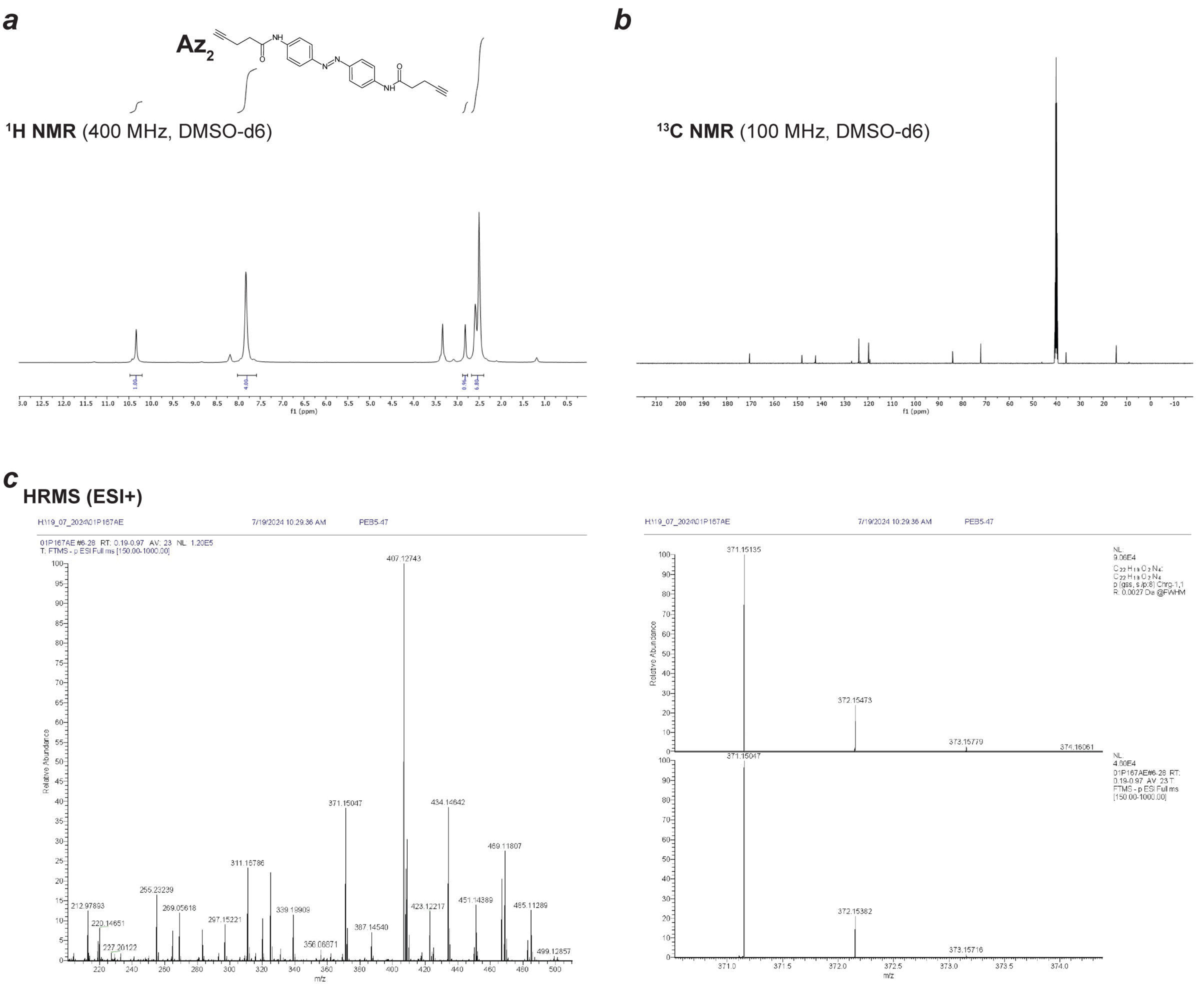


**Figure S14. Spectral data of compound Az$_2$: *a,* $^1$H NMR** (400 MHz, DMSO-*d$_6$*) $\delta$(ppm) 2.35-2.70 (m, **8H**), 2.82 (m, **2H**), 7.65-7.98 (m, **8H**), 10.34 (m, **2H**). ***b,* $^{13}$C NMR** (100 MHz, DMSO-*d$_6$*) $\delta$(ppm) 14.0 (**CH$_2$**), 35.3 (**CH$_2$**), 71.5 (**CH**), 83.5 (**C**), 119.2 (**CH**), 123.4 (**CH**), 141.8 (**C**), 147.6 (**C**), 169.8 (**C**). ***c,* HRMS (ESI)** *m/z* calc. for $C_{22}H_{19}N_4O_2$ [M-H]$^-$ 371.15135, found 371.15047.

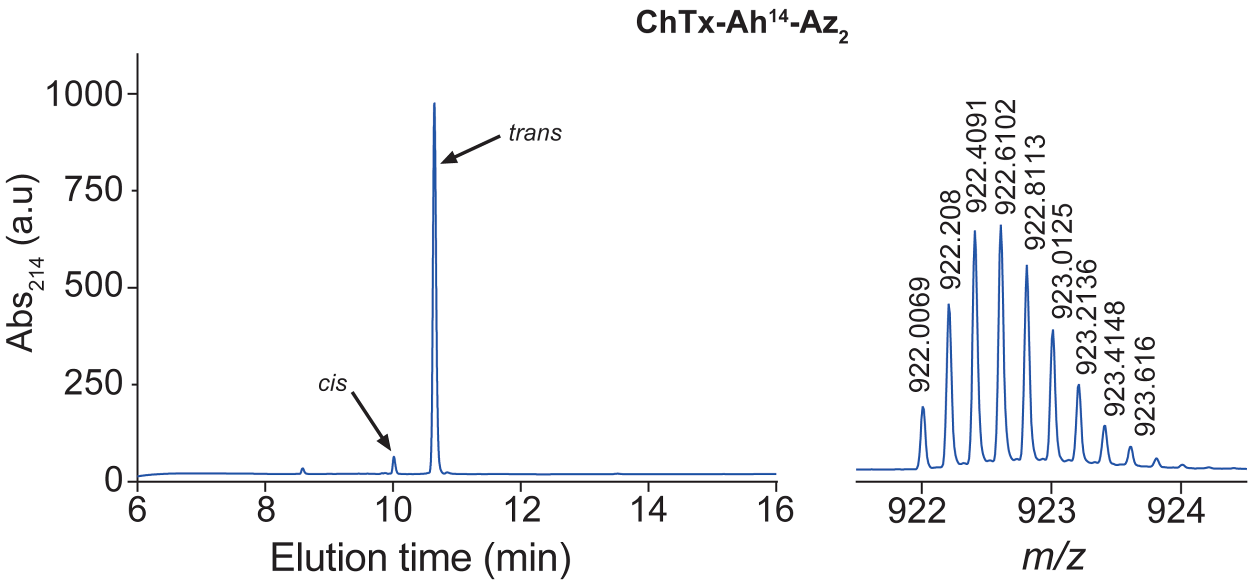


**Figure S15. Production and purification of the ChTx-Ah$^{14}$-Az$_2$.** Left panel: elution profile of ChTx-Ah$^{12}$-Az$_2$. Right panel: $[M + 4H]^{4+}$ MS. 3.6% *cis* configuration present (96.4% *trans* configuration).

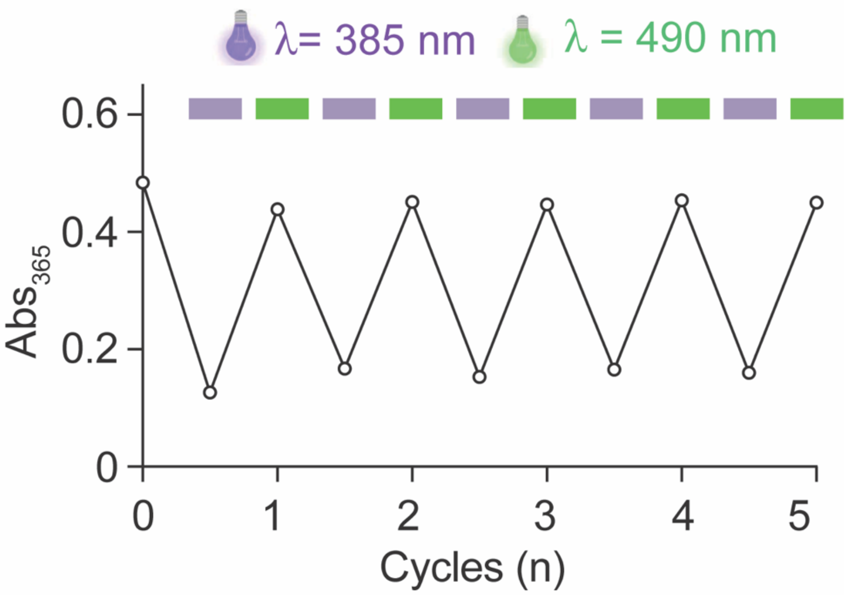


**Figure S16. Cycles of illuminations to probe ChTx-Ah$^{14}$-Az$_2$ fatigue.**

| | Chemical shifts ChTx-Ah[14]-Az$_1$ | Chemical shifts ChTx-Ah[14] | Chemical shift variations |
|---|---|---|---|
| PCA1 | 4.2 | 4.14 | 0.06 |
| F[2] | 4.44 | 4.43 | 0.01 |
| T[3] | 4.78 | 4.81 | -0.03 |
| N[4] | 4.9 | 4.92 | -0.02 |
| V[5] | 3.89 | 3.9 | -0.01 |
| S[6] | 4.98 | 5.01 | -0.03 |
| C[7] | 4.82 | 4.83 | -0.01 |
| T[8] | 4.39 | 4.41 | -0.02 |
| T[9] | 4.85 | 4.87 | -0.02 |
| S[10] | ND | ND | ND |
| K[11] | 4.03 | 4.02 | 0.01 |
| E[12] | 4.13 | 4.14 | -0.01 |
| C[13] | 4.62 | 4.64 | -0.02 |
| W[14] | ND | ND | ND |
| S[15] | 4.2 | 4.16 | 0.04 |
| V[16] | 3.63 | 3.62 | 0.01 |
| C[17] | 4.33 | 4.33 | 0 |
| **Q[18]** | **3.87** | **3.73** | **0.14** |
| R[19] | 4.04 | 4.04 | 0 |
| L[20] | 4.10 | 4.07 | 0.03 |
| H[21] | 4.87 | 4.89 | -0.02 |
| N[22] | 4.64 | 4.64 | 0 |
| T[23] | 4.64 | 4.62 | 0.02 |
| **S[24]** | **4.55** | **4.62** | **-0.07** |
| **R[25]** | **4.44** | **4.66** | **-0.22** |
| **G[26]** | **5.21** | **5.31** | **-0.1** |
| **K[27]** | **4.59** | **4.66** | **-0.07** |
| C[28] | 4.8 | 4.82 | -0.02 |
| M[29] | 4.71 | 4.74 | -0.03 |
| N[30] | 4.25 | 4.27 | -0.02 |
| K[31] | 3.80 | 3.81 | -0.01 |
| K[32] | 5.23 | 5.25 | -0.02 |
| C[33] | 5.04 | 5.07 | -0.03 |
| R[34] | 4.73 | 4.73 | 0 |
| C[35] | 5.47 | 5.42 | 0.05 |
| Y[36] | ND | ND | ND |
| **S[37]** | **4.2** | **4.13** | **0.07** |

**Table S1:** NMR chemical shifts of the Hα protons of *trans* ChTx-Ah[14]-Az$_1$, and ChTx-Ah[14]. The largest chemical shift variations (≥ 0.07 ppm, in bold) have been colored in yellow in **Figure 5**. ND = Not determined.

**References**

[1] A. Banerjee, A. Lee, E. Campbell, R. Mackinnon, *Elife* **2013**, *2*, e00594.

[2] E. F. Pettersen, T. D. Goddard, C. C. Huang, G. S. Couch, D. M. Greenblatt, E. C. Meng, T. E. Ferrin, *J Comput Chem* **2004**, *25*, 1605-1612.

[3] aE. Esteve, S. Smida-Rezgui, S. Sarkozi, C. Szegedi, I. Regaya, L. Chen, X. Altafaj, H. Rochat, P. Allen, I. N. Pessah, I. Marty, J. M. Sabatier, I. Jona, M. De Waard, M. Ronjat, *J Biol Chem* **2003**, *278*, 37822-37831; bL. Lopez, S. De Waard, H. Meudal, C. Caumes, K. Khakh, S. Peigneur, B. Oliveira-Mendes, S. Lin, J. De Waele, J. Montnach, S. Cestele, A. Tessier, J. P. Johnson, M. Mantegazza, J. Tytgat, C. Cohen, R. Beroud, F. Bosmans, C. Landon, M. De Waard, *Biomed Pharmacother* **2023**, *165*, 115173; cL. Lopez, J. Montnach, B. Oliveira-Mendes, K. Khakh, B. Thomas, S. Lin, C. Caumes, S. Wesolowski, S. Nicolas, D. Servent, C. Cohen, R. Beroud, E. Benoit, M. De Waard, *Front Cell Dev Biol* **2021**, *9*, 798588.
